# Targeting tumor O‐glycosylation modulates cancer–immune‐cell crosstalk and enhances anti‐PD‐1 immunotherapy in head and neck cancer

**DOI:** 10.1002/1878-0261.13489

**Published:** 2023-07-24

**Authors:** Mei‐Chun Lin, Ya‐Ting Chuang, Hsin‐Yi Wu, Chia‐Lang Hsu, Neng‐Yu Lin, Min‐Chuan Huang, Pei‐Jen Lou

**Affiliations:** ^1^ Department of Otolaryngology National Taiwan University Hospital Taipei Taiwan; ^2^ Department of Medical Research National Taiwan University Hospital Taipei Taiwan; ^3^ Instrumentation Center National Taiwan University Taipei Taiwan; ^4^ Graduate Institute of Anatomy and Cell Biology, College of Medicine National Taiwan University Taipei Taiwan

**Keywords:** core 1 β1,3‐galactosyltransferase, head and neck cancer, IL‐6, immune checkpoint inhibitor, itraconazole, O‐glycosylation

## Abstract

Cells in the tumor microenvironment (TME) communicate via membrane‐bound and secreted proteins, which are mostly glycosylated. Altered glycomes of malignant tumors influence behaviors of stromal cells. In this study, we showed that the loss of core‐1 β1,3‐galactosyltransferase (C1GALT1)‐mediated O‐glycosylation suppressed tumor growth in syngeneic head and neck cancer mouse models. O‐glycan truncation in tumor cells promoted the M1 polarization of macrophages, enhanced T‐cell‐mediated cytotoxicity, and reduced interleukin‐6 (IL‐6) levels in the secretome. Proteasomal degradation of IL‐6 was controlled by the O‐glycan at threonine 166. Both IL‐6/IL‐6R blockade and O‐glycan truncation in tumor cells induced similar pro‐inflammatory phenotypes in macrophages and cytotoxic T lymphocytes (CTLs). The combination of the O‐glycosylation inhibitor itraconazole and anti‐programmed cell death protein 1 (anti‐PD‐1) antibody effectively suppressed tumor growth *in vivo*. Collectively, our findings demonstrate that O‐glycosylation in tumor cells governs their crosstalk with macrophages and CTLs. Thus, targeting O‐glycosylation successfully reshapes the TME and consequently enhances the efficacy of anti‐PD‐1 therapy.

## Graphical abstract

This study found that O‐glycan truncation induced proteasomal degradation of interleukin‐6 to suppress tumor growth by promoting M1 macrophage polarization and enhancing T cell‐mediated cytotoxicity in head and neck cancer *in vitro* and *in vivo*. Combining an O‐glycosylation inhibitor with anti‐programmed cell death protein 1 antibody effectively suppressed tumor growth, emphasizing the role of O‐glycosylation in modulating the tumor microenvironment.

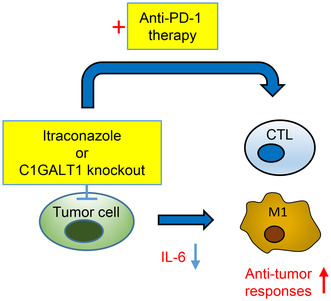

**Abbreviations**
anti‐PD‐1: anti‐programmed cell death protein 1
BMDM: bone marrow‐derived macrophage
C1GALT1: core‐1 β1,3‐galactosyltransferase
CM: conditioned media
cRNA: complimentary RNA
CTL: cytotoxic T lymphocyte
DMEM: Dulbecco's modified Eagle medium
ER: endoplasmic reticulum
ERAD: ER‐associated degradation
ETD: electron‐transfer dissociation
EThcD: electron‐transfer/higher‐energy collision dissociation
GBM: glioblastoma multiforme
GO: Gene Ontology
HCD: higher‐energy C‐trap dissociation
HNC: head and neck cancer
HRP: horseradish peroxidase
ICI: immune checkpoint inhibitor
LC–MS/MS: liquid chromatography with tandem mass spectrometry
PD‐1: programmed cell death protein 1
PD‐L1: programmed cell death ligand 1
RT: reverse transcription
SAMP: self‐associated molecular pattern
sgRNA: small guide RNA
TAM: tumor‐associated macrophages
TME: tumor microenvironment
VVA: *Vicia villosa* agglutinin

## 1 Introduction

Immune checkpoint inhibitors (ICIs) targeting CTLA‐4 and the PD‐1/PD‐L1 axis have shown unprecedented and durable responses in cancer patients by restoring the antitumor activities of T cells [1]. Head and neck cancer (HNC) is among the earliest cancer types that showed promising response rates to ICI therapy [2]. Although PD‐1 blockade has shown promising results in some patients with recurrent/metastatic HNC, most tumors remain resistant to ICI treatment [3, 4, 5]. Several mechanisms, including inadequate function of antitumor effector cells and increased infiltration of immune suppressive cells into the tumor microenvironment (TME), regulate the resistance to ICIs [6, 7, 8]. Therefore, the combination of ICIs with other immune‐modulating agents that eliminate immunosuppressive cells and reinvigorate exhausted T cells has been the focus of research to overcome ICI resistance [9].

Aberrant glycosylation in cancer is indispensable for the survival, proliferation, and dissemination of tumor cells during multistep tumorigenesis [10]. The immune response to tumors is a complex process influenced by many factors [11], and various strategies have been proposed to overcome immune resistance to tumors. The regulation of glycosylation is an interesting but unproven hypothesis that remains to be further investigated. Transformed epithelial cells utilize the built‐in glycosylation machinery to facilitate immune evasion. Although the overall glycome drifts away from its normal counterpart, tumor cells can disguise themselves as host‐like glycans [12, 13]. Moreover, tumor‐associated glyco‐epitopes, such as sialyl‐Tn, sialyl‐T, and sialyl‐Lewis, and Lewis antigens, interact with the surrounding cells to release immunosuppressive signals [14, 15]. Subsequently, the hijacked immune system allows or aids the growth and metastasis of the transformed tumor, thereby creating an immunosuppressive niche [16, 17].

Interleukin‐6 (IL‐6), a glycoprotein with 184 amino acids and a molecular weight of 23–30 kDa [18], is a multifunctional cytokine that regulates inflammation, immune responses, and hematopoiesis [19]. IL‐6 in the TME derives from tumor cells, myeloid cells, and fibroblasts [20]. Elevated serum IL‐6 levels have been detected in patients with advanced‐stage cancers, including HNC [21, 22]. Various reports have shown that IL‐6 is involved in the proliferation, survival, invasion, and metastasis of tumor cells [23]. Several glycoforms of IL‐6 have been reported [24]. However, the function of site‐specific N‐ or O‐glycans in IL‐6 remains unelucidated.

Core‐1 β1,3‐galactosyltransferase (C1GALT1) controls the bottleneck in GalNAc‐type O‐glycosylation. Its overexpression is associated with poor survival in patients with various human cancers, including HNC [25, 26], gastric cancer [27], pancreatic cancer [28], and liver cancer [29, 30]. Some studies have reported that the loss of C1GALT1 function can promote malignant properties in certain cancers [31, 32, 33], implying a tumor suppressive role of C1GALT1. These findings suggest that the function of C1GALT1 may vary depending on the type of cancer. In a previous study, we observed that C1GALT1 regulates ligand‐binding and phosphorylation of EGFR and enhances metastasis of HNC tumors [26], indicating the tumor‐promoting role of C1GALT1 in HNC. Here, we evaluated the role of C1GALT1‐mediated O‐glycosylation in the crosstalk between tumor cells and immune cells. In a syngeneic mouse model, we found that the loss of C1GALT1 in HNC cells suppressed tumor growth. C1GALT1‐mediated O‐glycosylation in HNC cells regulated differentiation of macrophages and activation of cytotoxic T lymphocytes (CTLs) by controlling proteasomal degradation of IL‐6. We previously reported that itraconazole, an anti‐fungal agent, can inhibit C1GALT1 expression [26]. In this study, the combination of itraconazole and anti‐programmed cell death protein 1 (anti‐PD‐1) antibody effectively inhibited tumor growth *in vivo*. Altogether, the current work unravels the role of C1GALT1‐mediated O‐glycosylation in modulating the TME. Targeting cancer‐associated O‐glycosylation via C1GALT1 inhibition could be an attractive strategy to enhance anti‐PD‐1 immunotherapy effects.

## 2 Materials and methods

### 2.1 Cell culture, transfection, and generation of C1GALT1‐knockout cells

MTC‐Q1 cells were a gift from K.‐W. Chang (Academia Sinica) [34]. SAS (RRID:CVCL_1675) and OEC‐M1 (RRID:CVCL_6782) cells were a gift from J.‐S. Chia (National Taiwan University). THP‐1 (RRID:CVCL_0006), Jurkat (RRID:CVCL_0065), FaDu (RRID:CVCL_1218), and SCA9 cells were purchased from the American Type Culture Collection, Manassas, VA, USA. All cell lines were authenticated by STR DNA profiling analysis. The culture medium for SAS, FaDu, SCA9, and MTC‐Q1 cells was Dulbecco's modified Eagle medium (DMEM) supplemented with 10% FBS (Thermo Fisher Scientific, Waltham, MA, USA). For OEC‐M1, THP‐1, and Jurkat cells, the culture media was Roswell Park Memorial Institute (RPMI) 1640 supplemented with 10% FBS (Thermo Fisher Scientific). All experiments were performed with mycoplasma‐free cells.

For C1GALT1 knockdown, the cells were transfected with 20 nm siRNA against *C1GALT1* (siC1GALT1‐1: 5′‐UUAGUAUACGUUCAGGUAAGGUAGG‐3′, siC1GALT1‐2: 5′‐UUAUGUUGGCUAGAAUCUGCAUUGA‐3′) using Lipofectamine RNAiMAX (Invitrogen, Waltham, MA, USA). A nontargeting siRNA (siCtr, 5′‐CAACCUCAGCCAUGUCGACUGGUUU‐3′, 5′‐AAACCAGUCGACAUGGCUGAGGUUG‐3′) was used as the control.

To knock out C1GALT1, we used the CRISPR/Cas9 system. The small guide RNA (sgRNA) targeting *C1GALT1* was designed according to database predictions. The target sequences for human and mouse were 5′‐GCAACACTTTGTTACAACGC‐3′ and 5′‐GAGTATTTTGTTGCGAGAAGAGG‐3′, respectively. Two plasmids were used: The first is a pAll‐Cas9.Ppuro plasmid (National RNAi Core Facility at Academia Sinica, Taiwan) containing Cas9 and sgRNA of *C1GALT1*. The second plasmid is a pSurrogate reporter plasmid containing a sgRNA target sequence located between an in‐frame EGFP cassette and an out‐of‐frame mCherry cassette. Briefly, SAS, SCA9, and MTC‐Q1 cells at a density of 1 × 10^6^ cells per well in a six‐well plate were cotransfected with 2.5 μg of pAll‐Cas9.Ppuro and 2.5 μg of pSurrogate reporter plasmids using the Lipofectamine 3000 kit (Invitrogen). At 48 h after transfection, viable mCherry‐positive cells were sorted using a 4‐laser FACSAriaIII sorter (BD Biosciences, Franklin Lakes, CA, USA) and then cultured in 96‐well plates as single cells. Single colonies with successful *C1GALT1* knockout were confirmed by DNA sequencing and western blotting. Clones without *C1GALT1* knockout were used as the control (Mock) [35].

### 2.2 Animals

All animal experiments were approved by the Institutional Animal Care and Use Committee of the National Taiwan University College of Medicine (Taipei, Taiwan; no. 20200014). Five‐week‐old female mice were obtained from the National Laboratory Animal Center, Taipei, Taiwan. For orthotopic models, SCA9 (5 × 10^5^) and MTC‐Q1 cells (1 × 10^6^) were injected into the tongues of Swiss Webster and C57BL/6 mice, respectively. For the subcutaneous model, MTC‐Q1 cells (2 × 10^6^) were injected subcutaneously into the flanks of C57BL/6 mice. Mice were sacrificed when the tumor volume exceeded 20 mm or the body weight was reduced by ≥ 20%. To evaluate the effect of itraconazole and anti‐PD‐1 antibody treatment on tumor growth in a subcutaneous model, itraconazole (600 μg/mouse, Sigma Aldrich, St. Louis, MO, USA) or the solvent control was administered intraperitoneally every day. The IgG2a isotype (clone 2A3) and anti‐PD‐1 (clone RMP1‐14) controls were obtained from Bio X Cell (Lebanon, NH, USA) and intraperitoneally injected twice a week (300 μg/mouse).

### 2.3 AlamarBlue™ assays

Cells (3 × 10^3^) were seeded into one well of a 96‐well plate. At 72 h, alamarBlue™ reagent (10 μL/well) was added to culture media, and absorbance was detected at 570 and 600 nm 2 h later. To evaluate the effect of itraconazole on cell viability, DMSO or itraconazole was added at 24 h and cell viability was measure at 72 h.

### 2.4 Immunohistochemistry

Paraffin‐embedded tissues were incubated overnight with the primary antibodies at 4 °C. The UltraVision™ Quanto Detection System (Thermo Fisher Scientific) was used, and signals were visualized using the DAB Quanto Chromogen provided in the same kit. All the sections were counterstained with hematoxylin. For assessing the intensity, each field was scored from 0 to 3. The primary antibodies used were anti‐mouse MRC1, Granzyme B, CD4, CD8, FoxP3 (Cell Signaling Technology, Danvers, MA, USA), anti‐human C1GALT1 (Santa Cruz Biotechnology, Dallas, TX, USA), CD8, and MRC1 (Cell Signaling Technology). Details of antibodies were listed in Table S1.

### 2.5 ELISArray

Blood collection was performed on the same day as animal sacrifice, for tumor harvesting, and stored until the experiments. Mouse Inflammatory Cytokines Multi‐Analyte ELISArray was performed according to the manufacturer's protocol (QIAGEN, Hilden, Germany). Briefly, serum samples (1 : 25) were incubated in 96‐well plates coated with specific antibodies against each cytokine for 2 h at room temperature. Then, detection antibodies and avidin‐horseradish peroxidase (HRP) were added and incubated for 2 and 1 h, respectively, at room temperature. The optical density (O.D.) at 450 nm was measured using an ELISA reader.

### 2.6 RNA extraction and real‐time RT‐PCR analysis

Total RNA was extracted using TRIzol reagent (Invitrogen) according to the manufacturer's instructions. Two micrograms of total RNA was used in reverse transcription (RT) reaction using the High‐Capacity cDNA Reverse Transcription Kit (Applied Biosystems, Waltham, MA, USA). The cDNA was subjected to real‐time PCR. Relative quantity of gene expression was normalized to *GAPDH* and analyzed with the mxpro Software (Stratagene, San Diego, CA, USA). To detect residual Cas9 in Mock or C1GALT1 KO cells, the sequences of primers were 5′‐CAGATTCGCCTGGATGACCA‐3′ and 5′‐ATCCGCTCGATGAAGCTCTG‐3′. Control cells were transiently transfected with pAll‐Cas9.Ppuro plasmid and harvested at 48 h. Detailed primer sequences were listed in Table S2.

### 2.7 Microarray and data analysis

Total RNA was extracted and used to produce Cyanine 3‐CTP‐labeled complimentary RNA (cRNA) using Agilent's Low Input Quick Amp WT Labeling Kit. cRNA was hybridized with the SurePrint G3 Human Gene Expression Microarrays v3 8X60K (Agilent p/n G4858A‐072363, Algilent Technologies, Santa Clara, CA, USA). Microarrays were scanned in an Agilent Microarray Scanner (Agilent G2505C) as reported in the guidelines from the manufacturer and the image analysis was made with Agilent Feature Extraction Software with parameters protocol: GE1_105_Dec08 and grid template: 072363_D_F_20150612. Microarray experiments were performed by Microarray Core Lab, Academia Sinica.

Raw transcriptome data (Accession number: GSE116418) were processed for background correction using the method “normexp” implemented in the r package limma and then normalized between arrays using quantile normalization. Differentially expression genes were determined using the r package limma with *P* < 0.05. Gene Ontology (GO) over‐representation analysis was performed on the differentially expressed genes by the enrichGO function in the r package clusterprofiler. Significantly enriched GO terms (*P* < 0.01) were constructed into an enrichment map and visualized by cytoscape (boston, ma, usa).

### 2.8 Bone marrow‐derived macrophage and THP‐1 cell coculture assays

Bone marrow cells were harvested from the femurs and tibias of Swiss Webster mice and incubated overnight in 10% FBS/DMEM. The floating cells were discarded, and the remaining attached cells were incubated with 10% FBS/20% L929 cells. On Day 7, bone marrow‐derived macrophages (BMDMs) were cocultured with tumor cells for 24 h before harvesting for differentiation marker analysis. THP‐1 cells were differentiated with PMA (5 ng·mL^−1^) for 24 h and subsequently cocultured with cancer cells for another 24 h before harvesting for the analysis of differentiation markers.

The blocking antibodies used were IgG2 isotype control (LTF‐2; Bio X Cell, Lebanon, NH, USA) and anti‐IL‐6R (15A7; Bio X Cell) for mouse cells and IgG1 isotype control and tocilizumab (Bio X Cell) for human cells. Macrophages were preincubated with blocking antibodies (20 μg·mL^−1^) for 30 min before being cocultured with tumor cells.

### 2.9 CTL assay

To generate primary CTLs, CD3^+^CD8^+^ cells were harvested from the splenocytes of Swiss Webster or C57BL/6 mice. CD3^+^CD8^+^ T cells were activated using CD3/CD28 Dynabeads and then cultured with IL‐2 (30 U·mL^−1^) for 1 week. For activation of Jurkat cells, PMA (100 ng·mL^−1^) and PHA‐L (5 μg·mL^−1^) were added to the culture medium for 48 h.

The activated CTLs mentioned above were used to kill cancer cells. To detect apoptosis, cancer cells were preincubated with Incucyte® Caspase‐3/7 dyes, which become fluorescent when cleaved by activated Caspase‐3/7. CTLs and cancer cells were cocultured at a ratio of 1 : 5 for 4–8 h, and fluorescent apoptotic cancer cells were then visualized using fluorescence microscopy.

In experiments using blocking antibodies, the CTLs were preincubated with antibodies for 30 min before coculturing. For mouse cells, the antibodies used were IgG2 isotype control (LTF‐2; Bio X Cell), anti‐IL‐6R (15A7; Bio X Cell), and anti‐PD‐1 (RMP1‐14; Bio X Cell). For human cells, the antibodies used were IgG1 isotype control, tocilizumab, and nivolumab (Bio X Cell).

Images of fluorescent cells were quantified using the imagej software (developed by the National Institute of Health, Bethesda, MD, USA, and the data were analyzed using Student's *t*‐test.

### 2.10 Immunofluorescence microscopy

Cells (5 × 10^3^) were seeded on poly‐lysine‐coated coverslips a day before the experiment. DMSO or Eeyarestatin I (10 μm) was added to the culture media for 8 h, and the cells were washed with PBS and then fixed with 4% paraformaldehyde for 15 min at room temperature. After rinsing, samples were incubated with 0.1% triton X‐100 in PBS for 10 min. After washing, samples were incubated with anti‐human IL‐6 antibody (R&D Systems, Minneapolis, MN, USA) overnight at 4 °C. The next day, samples were washed and incubated with fluorochrome‐conjugated secondary antibody for 1 h at room temperature. After being washed and stained with DAPI (1 μg·mL^−1^), samples were mounted and images were taken by fluorescence microscopy. Levels of IL‐6 were quantified using the imagej software.

### 2.11 LC–MS/MS analysis and database search

LC–MS/MS analysis was performed on a quadrupole‐ion trap Orbitrap Fusion Lumos Tribrid spectrometer (Thermo Fisher Scientific, San Jose, CA, USA) equipped with a nanospray ion source. The peptides were separated on an Ultimate 3000 nanoLC system (Thermo Fisher Scientific, Bremen, Germany). Peptide mixtures were loaded onto a C18 Acclaim PepMap NanoLC column (Thermo Scientific, San Jose, CA, USA; 75 μm inside diameter and 25 cm length) packed with 2 μm particles with a pore size of 100 Å. Mobile phase A was 0.1% formic acid in water, and mobile phase B consisted of 100% acetonitrile and 0.1% formic acid. A segmented gradient was performed over 90 min from 2% to 35% solvent B at a flow rate of 300 nL·min^−1^ and a column temperature of 35 °C. Mass spectrometry was performed in data‐dependent mode with full MS spectra (mass accuracy < 5 p.p.m. and resolution of 120 000 at *m/z* = 200). The normalized AGC was set to 125%, with a maximum injection time of 50 ms. High‐energy collision‐activated dissociation (HCD)‐MS/MS with a resolution of 15 000 was used to fragment multiply charged ions with higher intensity in 3 s within a 1.4 Da isolation window at a normalized collision energy of 32, and then the HCD‐product‐dependent ETD acquisition method (HCD‐pd‐ETD) was applied. HCD scans containing either 204.0867 *m/z* or 138.0545 *m/z* peaks within 15 p.p.m. of the top 20 most intense peaks triggered a separate EThcD scan (with 25% HCD supplemental activation) on the same precursor. MS2 scans were analyzed in the Orbitrap for both HCD and EThcD at a 15 000 resolution and 1.4 *m/z* isolation window, with a 100 ms injection time and a normalized AGC of 200% for HCD and a 250 ms injection time with a normalized AGC of 600% for EThcD.

Raw MS/MS data were searched against the UniProt knowledgebase‐reviewed (Swiss‐Prot) human protein database (downloaded in August 2020) using the mascot search algorithm via the proteome discoverer (PD) package (version 2.2; Thermo Scientific). The search parameters were set as follows: peptide mass tolerance, 10 p.p.m.; MS/MS ion mass tolerance, 0.02 Da; enzyme, trypsin; missed cleavages, ≤ 2; and variable modifications, namely oxidation on methionine, deamidation on asparagine and glutamine residues, HexNAx on serine or threonine, and carbamidomethylation of cysteine residues. The peptides were filtered using 1% false discovery rate.

### 2.12 Tissue samples

All human tissues (*n* = 87, from January 2016 to December 2018) were obtained according to a protocol approved by the institutional review board of the National Taiwan University Hospital, Taipei, Taiwan. The experiments were undertaken with the understanding and written consent of each subject. The IRB numbers are 201304078RIND and 201707038RINB. The study methodologies conformed to the standards set by the Declaration of Helsinki.

### 2.13 Detection of human and mouse serum IL‐6

The human IL‐6 ELISA (R&D Systems) and mouse IL‐6 ELISA (BioLegend, San Diego, CA, USA) were performed according to the manufacturer's protocol. Briefly, standards and sera (100 μL for human and 50 μL for mouse) per well was added to an array plate, which was coated with a monoclonal antibody against IL‐6, and incubated for 2 h at room temperature. The plate was then washed and incubated with an HRP‐conjugated polyclonal antibody against IL‐6 for 2 h at room temperature. Next, the plate was incubated with the substrate solution for 20 min at room temperature. After the addition of stop solution, the absorbance was measured at 450 nm, and IL‐6 concentration was calculated using the standards as calibrators.

### 2.14 Human cytokine array, western blot analysis, and lectin pull‐down assay

SAS cells (2 × 10^7^) were cultured in serum‐free DMEM for 24 h before harvesting the conditioned medium (CM). The proteins from CM were quantified (150 μg), and a human cytokine array (R&D Systems) was performed according to the manufacturer's protocol.

Proteins from the tissue, cell lysates, or CM were separated via SDS/PAGE and transferred onto a PVDF membrane, which was then blocked in 5% nonfat milk for 1 h at room temperature and incubated with primary antibodies against C1GALT1, MHC I, GAPDH (Santa Cruz Biotechnology), PD‐L1, IL‐1α, MCP‐1, IL‐6, IL‐8, STAT3 and phospho‐STAT3 (Cell Signaling Technology), CD155 (R&D Systems) at 4 °C overnight. The complete list of antibodies was in Table S1. HRP‐conjugated secondary antibodies (Jackson ImmunoResearch, West Grove, PA, USA) were used to detect the protein bands.

For the lectin pull‐down assay, 250 μg of total proteins from CM was incubated with *Vicia villosa* agglutinin (VVA)‐conjugated beads (Vector Laboratories, Burlingame, CA, USA) for 18 h at 4 °C with constant rotation. The washed beads were boiled at 95 °C for 10 min, and VVA binding proteins were evaluated via western blot analysis.

### 2.15 Tissue sample preparation, tumor‐infiltrating leukocytes and flow cytometry

MTC‐Q1 tumors were dissociated into single cells using GentleMACS tissue dissociator and its kit (Miltenyi Biotec, Gladbach, Germany). Single cells were washed with PBS and incubated with TruStain FcX (BioLegend, San Diego, CA, USA) for 15 min on ice. The cells were incubated with desired antibodies (Table S1) for 30 min on ice, protected from light. The single cells were washed with PBS and resuspended with FACS buffer for flow cytometry analysis. Samples containing 1 × 10^5^ single cells were collected on a BD LSRFortessa (BD Biosciences) with facsdiva software (BD Biosciences), and the data were analyzed using flowjo (Tree Star, Ashland, OR, USA).

Tumor‐infiltrating leukocytes were defined as CD45^+^, tumor‐associated macrophages (TAM) as F4/80^+^, lymphocytes as CD3^+^, M2 macrophages as CD206^+^F4/80^+^, tumor‐reactive effector T cells as CD279^+^CD44^+^CD8^+^CD3^+^.

### 2.16 Statistical analysis

All experiments were performed at least in triplicate and data were represented as mean ± standard error of the mean (SEM) or mean ± standard deviation (SD). Two groups were compared using Student's *t*‐test. Numeric data were evaluated by one‐way ANOVA analysis. Linear correlation between two variables were evaluated using Pearson's correlation test. Nonparametric correlation were evaluated using Spearman's rank correlation analysis. Mann–Whitney *U* test was used for nonparametric data from animal and human samples. Statistical significance was set at *P* < 0.05.

## 3 Results

### 3.1 O‐glycan truncation via C1galt1 knockout suppresses tumor growth in syngeneic mouse models

**Fig. 1:**
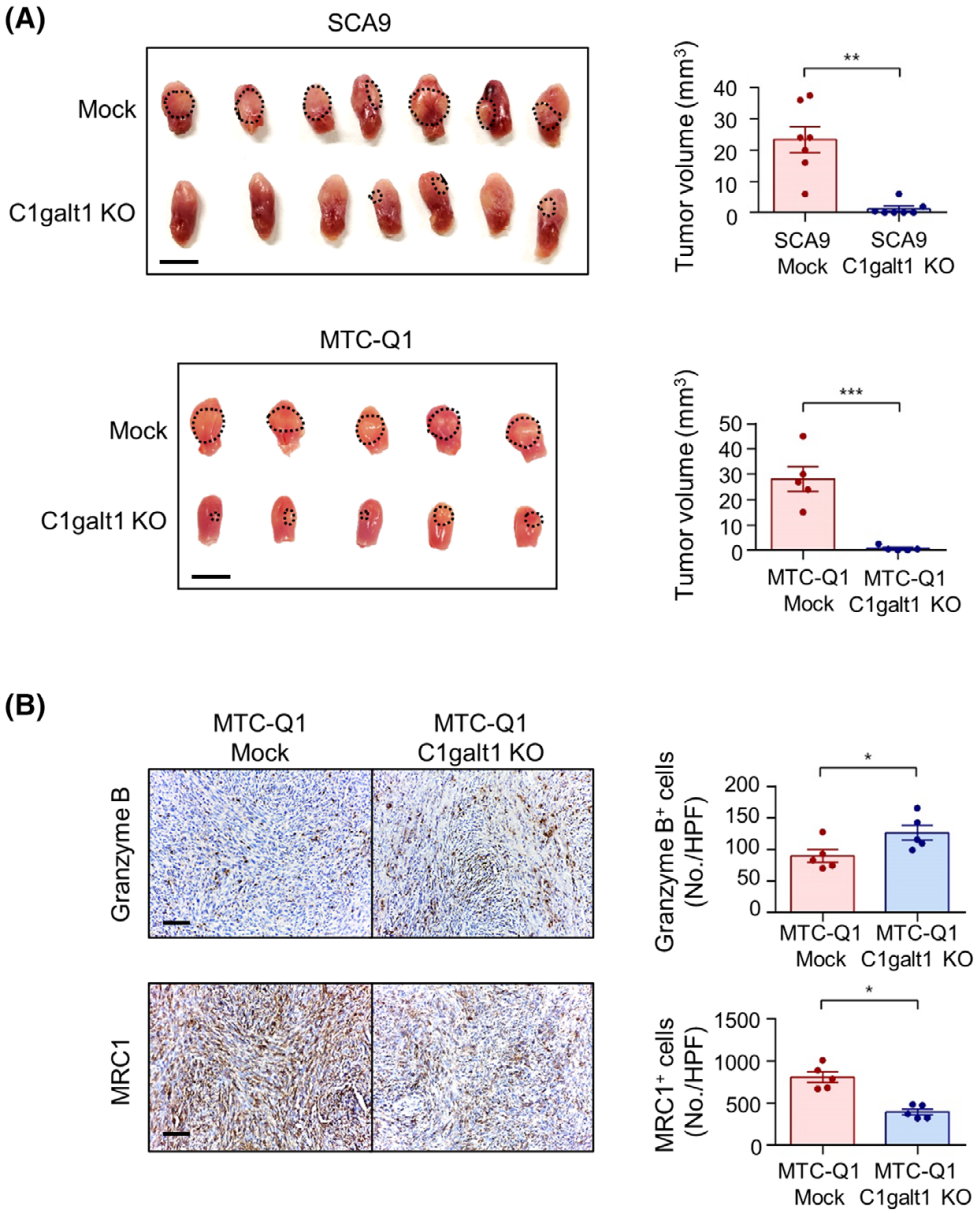
Effects of O‐glycan truncation on tumor growth and microenvironment in syngeneic mouse models. (A) C1GALT1 knockout suppressed tumor growth in syngeneic mouse models. SCA9 and MTC‐Q1 cells were orthotopically injected into the tongues of Swiss Wester (*n* = 7 for each group) and C57BL/6 (*n* = 5 for each group) mice, respectively. Mice were sacrificed 10 days after tumor inoculation. Images of whole tongues are shown at the left. Dashed circles indicate tumor regions. Scale bars, 5 mm. Right, volumes of Mock and C1galt1 knockout (KO) tumors are presented as mean ± SEM. **P < 0.01 and ***P < 0.001, analyzed using Student's *t*‐test. (B) Tumor‐infiltrating Granzyme B^+^ and MRC1^+^ cells. Granzyme B and MRC1 expression was analyzed using immunohistochemistry (IHC). Left, representative images of Granzyme B or MRC1 expression in Mock or C1galt1 KO tumors. Scale bars, 50 μm. Right, Statistical analysis of tumor‐infiltrating Granzyme B^+^ or MRC1^+^cells (*n* = 5 for each group). The number of positive cells was counted under high‐power fields of a microscope. At least three fields were taken from one tumor. Data are presented as mean ± SD. *P < 0.05 using a two‐tailed Student's *t*‐test.

C1GALT1 controls the bottleneck of GalNAc‐type O‐glycosylation, and C1GALT1 knockout is commonly used to study the effects of O‐glycan ablation [33]. Mock or C1galt1 knockout SCA9 and MTC‐Q1 cells were orthotopically injected into Swiss Webster and C57BL/6J mice, respectively. C1galt1 knockout almost completely blocked tumorigenicity compared with the control *in vivo* (Fig. 1A). However, the inhibition of cell viability by C1galt1 knockout *in vitro* was less remarkable (Fig. S1A). C1galt1 knockout cells had a slight increase in apoptosis compared with Mock cells (Fig. S1B). Owing to this salient difference, we suspected augmented immune responses in C1galt1 knockout tumors. To exclude the possibility that the residual Cas9 expression was causing our cells to be more immunogenic, we performed real‐time RT‐PCR and the results showed no detectable levels of *Cas9* expression in both Mock and C1GALT1 knockout cells (Fig. S2A). To identify the specific immune cells regulated via tumor O‐glycosylation, a panel of immune markers in Mock or C1galt1 knockout tumors was analyzed (Fig. 1B and Fig. S2B). A significantly increased number of Granzyme B^+^ cells and reduced number of MRC1^+^ cells were noted in C1galt1 knockout tumors compared with those in Mock tumors, indicating the involvement of cytotoxic immune cells and macrophages. Overall, these results suggest that O‐glycan truncation suppresses tumor growth *in vivo* and antitumor immunity may play a key role in this effect.

### 3.2 O‐glycan truncation in tumor cells promotes the M1 polarization of macrophages and enhances the activities of CTLs *ex vivo*

**Fig. 2:**
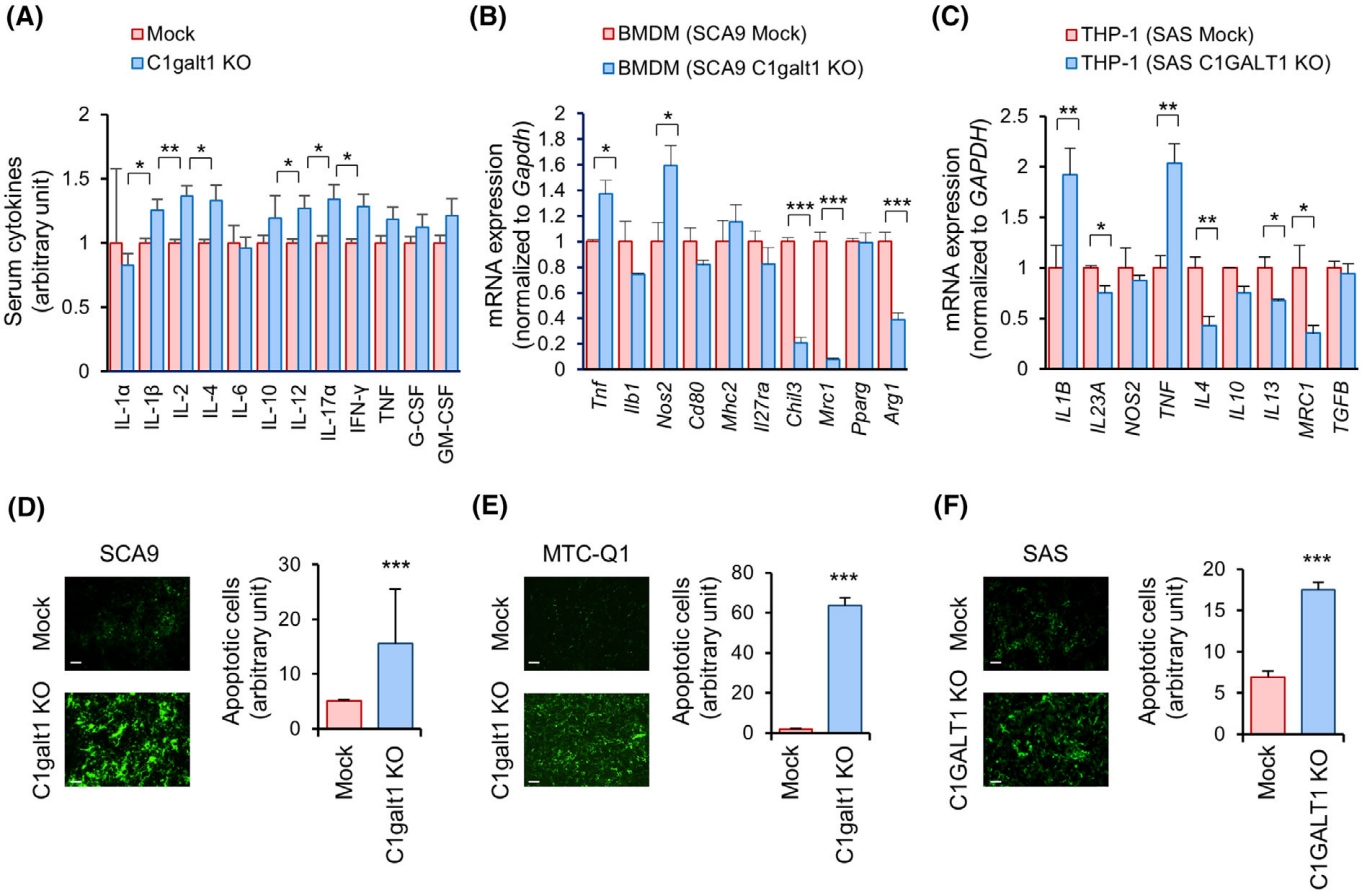
Effects of tumor cells with truncated O‐glycans on macrophages and cytotoxic T cells *ex vivo*. (A) Serum cytokine levels in Swiss Webster mice bearing Mock or C1galt1 knockout (KO) SCA9 tumors. Blood samples were collected before mice were sacrificed. Serum cytokine levels were measured using an ELISArray kit and data are presented as mean ± SD in arbitrary units (*n* = 3 mice/group). *P < 0.05 and **P < 0.01, analyzed using Student's *t*‐test. (B, C) Real‐time RT‐PCR analysis of macrophage differentiation markers. Primary BMDMs of Swiss Webster mice were differentiated into M0 macrophages using L929 conditioned medium for 7 days. THP‐1 cells were differentiated into macrophages via incubation with 5 μg·mL^−1^ PMA for 24 h. Macrophages were then cocultured with SCA9 or SAS cells in transwells for another 24 h. Data are presented as mean ± SD. *P < 0.05, **P < 0.01, and ***P < 0.001, analyzed using a two‐tailed Student's *t*‐test. Experiments were performed in triplicate. (D–F) Effects of O‐glycan truncation on the CTL‐induced apoptosis of cancer cells. Mouse primary CD3^+^/CD8^+^ cells were activated into CTLs with CD3/CD28 Dynabeads and mIL‐2 for 7 days. Human Jurkat cells were activated with 5 μg·mL^−1^ PHA‐L and 100 ng·mL^−1^ PMA for 24 h. The IncuCyte® was added to culture media 24 h before cancer cells were cocultured with CTLs. The ratio of effector:target was 1 : 5. Fluorescence intensity was quantified using the imagej software. Scale bars, 50 μm. Data are presented as mean ± SD in arbitrary units. ***P < 0.001, analyzed using a two‐tailed Student's *t*‐test. Experiments were performed in triplicate.

To understand the immune responses elicited by O‐glycan truncation, cytokines in the mouse serum were analyzed using ELISArray. The results showed that IL‐1β, IL‐2, IL‐4, IL‐12, IL‐17α, and IFN‐γ levels were significantly increased in mice harboring the C1galt1 knockout tumors (Fig. 2A). Next, the expression of macrophage differentiation markers in primary BMDMs cocultured with tumor cells was assessed using real‐time RT‐PCR analysis. Elevated expression of *Tnf* and *Nos2* and reduced expression of *Chil3*, *Mrc1*, and *Arg1* were observed in the BMDMs cocultured with C1galt1 knockout tumor cells, compared with those in the Mock tumor cells (Fig. 2B), suggesting that O‐glycan truncation in tumor cells induced the M1 polarization of cocultured BMDMs. Consistently, the THP‐1 cells cocultured with C1GALT1 knockout SAS cells also expressed significantly high *IL1B* and *TNF* and low *IL4*, *IL13*, and *MRC1* levels (Fig. 2C). Consistent with the effect of C1galt1 knockout, the BMDMs cocultured with itraconazole‐treated tumor cells were also M1 polarized (Fig. S3).

As an increase was observed in the number of tumor‐infiltrating Granzyme B^+^ cells in C1galt1 knockout tumors, we further performed *ex vivo* CTL assays to elucidate whether the T cell‐mediated cytotoxicity was involved. Increased CTL‐induced apoptosis was noted in C1galt1 knockout tumor cells compared with that in the Mock cells (Fig. 2D,E). Consistently, apoptosis was increased in the C1GALT1 knockout SAS cells (Fig. 2F). These results suggest that O‐glycan truncation in tumor cells enhances the T cell‐mediated cytotoxicity.

Consistent with the abovementioned results, the gene expression profile and functional analysis using control and C1GALT1 knockdown SAS cells showed that C1GALT1 regulated the pathways important for immune responses, such as T cell differentiation and activation, IL‐1 production, and TNF production (Fig. S4).

Overall, O‐glycan truncation by C1GALT1 knockout in tumor cells skewed the macrophages and CTLs toward antitumorigenic phenotypes.

### 3.3 C1GALT1 controls the proteasomal degradation of IL‐6

**Fig. 3:**
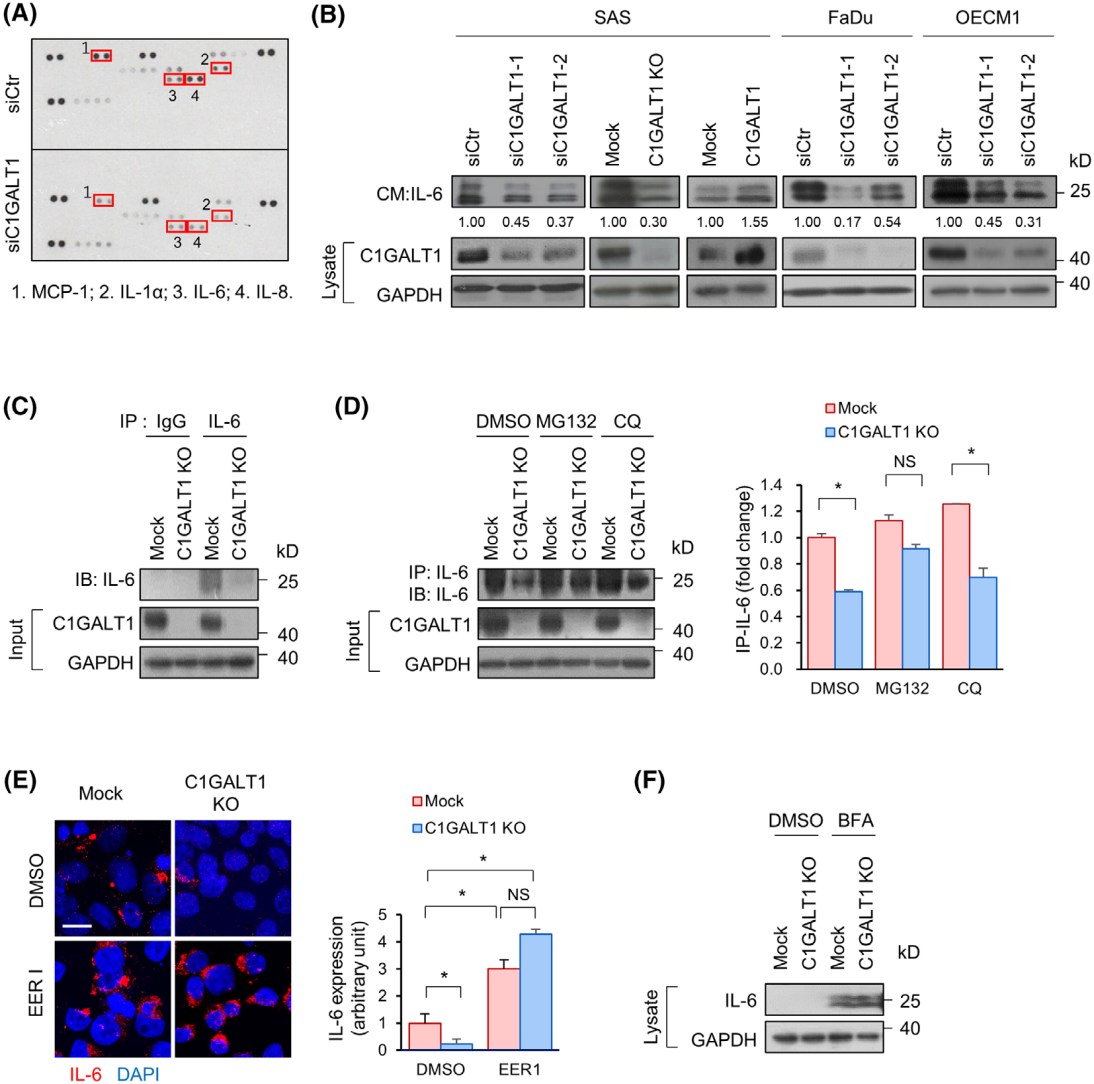
Effects of O‐glycosylation on the proteasomal degradation of IL‐6. (A) Cytokines regulated by C1GALT1. Cytokine levels in CM from SAS cells transfected with non‐targeting siRNA (siCtr) or siRNAs against *C1GALT1* (siC1GALT1) were evaluated using a Human Cytokine Array. Red rectangles indicate cytokines with differential expression. This experiment was performed once. (B) IL‐6 levels in HNC cells. C1GALT1 knockdown, knockout, or overexpression in SAS, FaDu, and OECM1 cells, as indicated, were used. IL‐6 expression in the CM was evaluated using western blot analysis. Representative images were shown. The intensity of IL‐6 bands (both isoforms) was quantified using the imagej software and the number was shown as indicated. GAPDH was used as an internal control. Experiments were performed in triplicate. (C) Expression of IL‐6 in cells. The lysates of Mock or C1GALT1 knockout (KO) SAS cells were incubated with isotype mouse IgG or mouse anti‐IL‐6 antibody at 4 °C overnight. The immunoprecipitated proteins were captured by protein G agarose beads and then immunoblotted (IB) with a rabbit anti‐IL‐6 antibody. Experiments were performed in triplicate. (D) Degradation pathways of IL‐6. Left panel, SAS cells were treated with DMSO, 20 μm MG132, or 10 μm chloroquine (CQ) for 12 h before harvesting them for immunoprecipitation (IP). Right panel, IL‐6 expression was quantified using the imagej software. All values were normalized to IL‐6 from Mock SAS cells which were treated with DMSO (first lane). Experiments were performed in triplicate and the data are presented as mean ± SD. *P < 0.05; NS, not significant, analyzed using a two‐tailed Student's *t*‐test. (E) Degradation of IL‐6 through ERAD. SAS cells were treated with 10 μm Eeyarestatin I (EER I) for 8 h before harvesting them for immunofluorescence microscopy. Left panel, representative images of IL‐6 expression (red) under a confocal microscope. Cell nuclei were counterstained with DAPI (blue). Right panel, IL‐6 signals were quantified using the imagej software. Data are presented as mean ± SD. *P < 0.05, NS, not significant, analyzed using a two‐tailed Student's *t*‐test. Experiments were performed in triplicate. (F) Western blot analysis of IL‐6 in CM and the lysates of Mock and C1GALT1 KO SAS cells treated with DMSO or 10 μg·mL^−1^ brefeldin A (BFA) for 12 h. GAPDH was used as an internal control. Experiments were performed in triplicate.

Several important surface molecules, including CD155, PD‐L1, and MHC I, influence the activation of the immune cells. Therefore, we examined the expression and O‐glycosylation of these molecules, but we found no differential expression or O‐glycosylation changes between Mock and C1GALT1 KO cells (Fig. S5A), suggesting that these molecules may not be directly responsible for C1GALT1‐mediated effects. Moreover, we used a transwell coculture system to evaluate macrophage differentiation. These results indicate that the behaviors of macrophages and CTLs were more likely influenced by secreted factors, such as cytokines and chemokines. We performed a human cytokine array, and our results showed that C1GALT1 knockdown in SAS cells reduced the expression of MCP‐1, IL‐1α, IL‐6, and IL‐8 compared with that in the control cells (Fig. 3A). Furthermore, western blot analysis showed that the expression of IL‐1α, IL‐6, and IL‐8 was reduced in the C1GALT1 knockout cells (Fig. S5B). We also analyzed the mRNA expression of *MCP‐1*, *IL‐1α*, *IL‐6*, and *IL‐8*. Interestingly, *IL‐6* expression did not correlate with the protein levels, suggesting that post‐translational modifications may regulate the IL‐6 expression (Fig. S5C). Therefore, we analyzed the effect of C1GALT1 overexpression, knockdown, and knockout on IL‐6 levels in different HNC cell lines. The results showed that while C1GALT1 knockdown or knockout reduced IL‐6 expression, C1GALT1 overexpression increased IL‐6 expression (Fig. 3B). We noticed that there were two forms of IL‐6 present in our cells. To clarify this, we performed western blot analysis of standard IL‐6 and endogenous IL‐6 from SAS cells (Fig. S5D). The results showed that both forms were IL‐6, and the difference in molecular weight may be due to glycosylation changes. Consistently, itraconazole, which inhibited C1GALT1 expression, significantly reduced the expression of IL‐6 in human and mouse cells (Fig. S5E). Real‐time RT‐PCR analysis showed that the effects of C1GALT1 on IL‐6 protein levels did not result from the changes in *IL6* mRNA levels (Fig. S5F). In addition, ectopic expression of IL‐6‐HA was lower in the C1GALT1 knockout 293FT cells than in the wild‐type cells (Fig. S5G). Overall, these results indicate a post‐translational regulation of IL‐6 expression by C1GALT1.

The extracellular levels of IL‐6 are controlled by IL‐6 synthesis, secretion, degradation, and receptor‐mediated endocytosis [19, 36]. The intracellular IL‐6 levels were analyzed via immunoprecipitation. Results showed that the intracellular levels of IL‐6 were reduced in C1GALT1 knockout cells (Fig. 3C). IL‐6 is synthesized and folded in the endoplasmic reticulum (ER) and then transported to the Golgi apparatus, where O‐glycosylation takes place. Post‐ER quality control can be mediated by proteasomal or lysosome degradation [37]. Western blot analysis showed that MG132 treatment, but not chloroquine, recovered intracellular IL‐6 levels in C1GALT1 knockout cells compared with those in Mock cells. (Fig. 3D). Golgi proteins targeted for proteasomal degradation undergo retrograde transport to the ER for ER‐associated degradation (ERAD). Eeyarestatin I, an ERAD inhibitor, successfully rescued the expression of IL‐6 in C1GALT1 knockout cells and obscured the difference in IL‐6 expression between Mock and C1GALT1 knockout cells (Fig. 3E). Treatment with brefeldin A, which inhibits retrograde transport from the Golgi apparatus to the ER, increased the intracellular expression of IL‐6 and alleviated the differential expression of IL‐6 between Mock and C1GALT1 knockout SAS cells (Fig. 3F). In summary, our results show that C1GALT1 regulates the expression of IL‐6 by controlling its proteasomal degradation via the ERAD pathway.

### 3.4 C1GALT1‐mediated O‐glycosylation at T166 of IL‐6 regulates IL‐6 stability

**Fig. 4:**
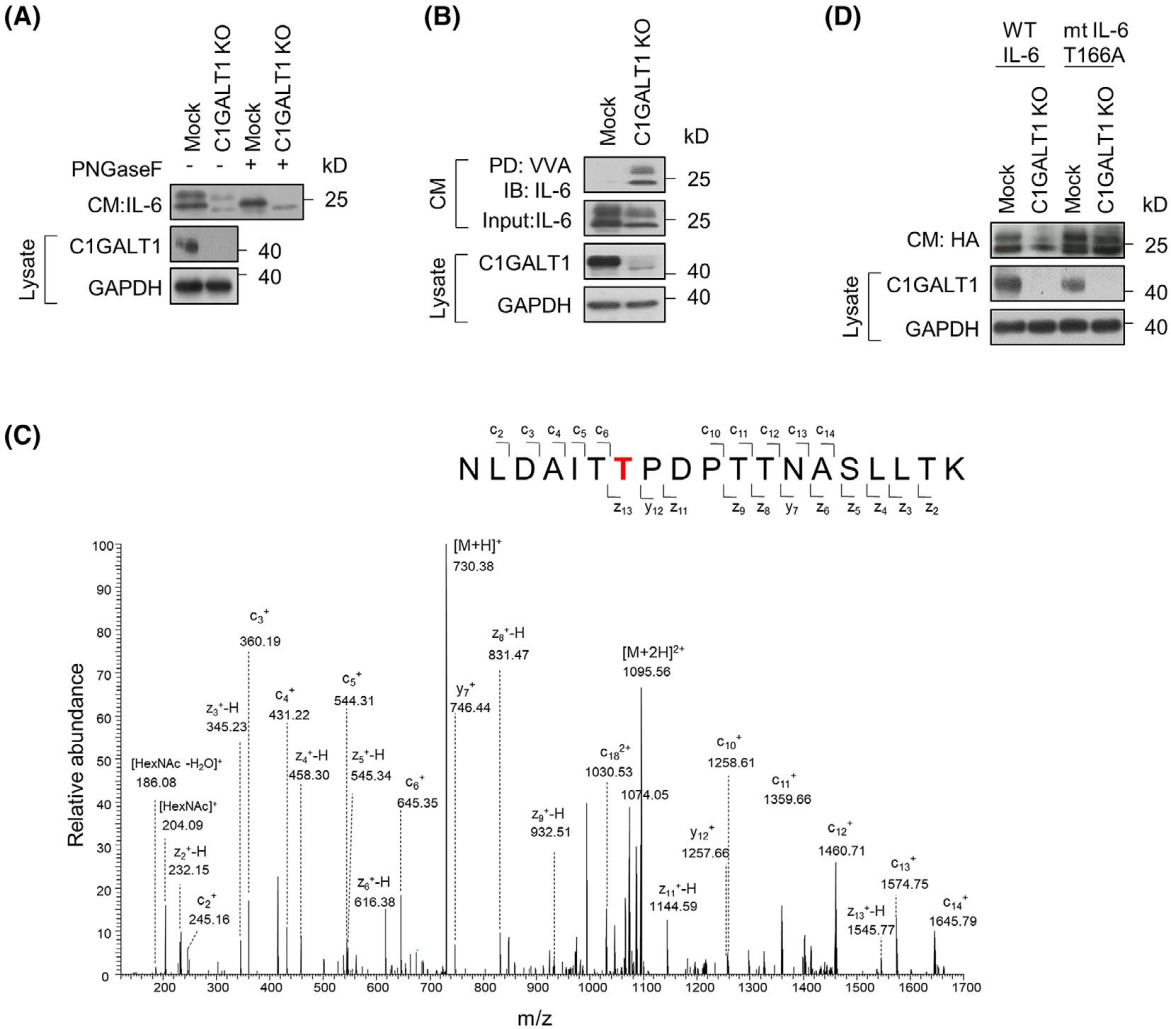
IL‐6 stability controlled by the O‐glycan at T166. (A) Decrease in the molecular weight of IL‐6 upon O‐glycan truncation. Western blot analysis of IL‐6 in CM from Mock or C1GALT1 knockout (KO) SAS cells treated with or without PNGaseF (1000 units·mL^−1^). GAPDH was used as an internal control. Experiments were performed in triplicate. (B) VVA lectin pull‐down assay of IL‐6. The IL‐6 in CM of Mock or C1GALT1 KO SAS cells was pulled down (PD) with VVA‐conjugated agarose beads for 18 h and then detected using western blot analysis. IL‐6 in the CM is shown in the upper panel. C1GALT1 and GAPDH in the cell lysates were shown in the lower panel. Experiments were performed in triplicate. (C) Tandem mass spectrum of the O‐glycosylated peptide NLDAITT(166)PDPTTNASLLTK of IL‐6. HA‐tagged IL‐6 was overexpressed in the C1GALT1 KO SAS cells. IL‐6 in the CM was purified using anti‐HA agarose beads. A HexNAc was identified on IL‐6 at T166. One batch of sample was analyzed. (D) Expression of mt IL‐6 T166A in SAS cells. Mock or C1GALT1 KO SAS cells were transfected with HA‐tagged wild‐type (WT) IL‐6 or HA‐tagged mutant IL‐6 (mt IL‐6 T166A) for 48 h. IL‐6 in CM was analyzed using western blotting. C1GALT1 and GAPDH in cell lysates were shown. Experiments were performed in triplicate.

According to the previous literature, the molecular weight of human IL‐6 ranged from 23 to 30 kD, including a 25‐kD O‐glycosylated form and a 30‐kD O‐ and N‐glycosylated form [38]. Although O‐glycans were known to exist on IL‐6, their specific functions and sites remain unclear [24]. We showed that C1GALT1 knockout led to a decrease in the molecular weight of IL‐6 compared with Mock cells, and the removal of N‐glycans by PNGase F did not eliminate the molecular weight differences (Fig. 4A). The result suggested that IL‐6 was O‐glycosylated by C1GALT1. Consistently, a lectin pull‐down assay showed increased binding of VVA to IL‐6 in C1GALT1 knockout cells compared with that in Mock cells, indicating that O‐glycan elongation on IL‐6 was regulated by C1GALT1 (Fig. 4B). The IL‐6 purified from C1GALT1 knockout SAS cells was subjected to LC–MS/MS analysis using the electron‐transfer/higher energy collision dissociation (EThcD) fragmentation method. A short O‐glycan was identified on the peptide sequence NLDAITTPDPTTNASLLTK. The precursor ion (*m/z* 730.377, 3+) with fragment ions (c_6_^+^, c_10_^+^, z_11_^+^, y_12_^+^, and z_13_^+^) confirmed a HexNAc modification at threonine (T) 166 (Fig. 4C). The same O‐glycosite was also identified in 293FT cells (Fig. S6). T166A mutation restored the protein levels of IL‐6 in C1GALT1 knockout cells (Fig. 4D). Overall, these results show that C1GALT1‐mediated O‐glycosylation at T166 regulates the stability of IL‐6.

### 3.5 Expression of C1GALT1 positively correlates with phospho (p)‐STAT3 levels in tumors and IL‐6 levels in the sera of patients with HNC

**Fig. 5:**
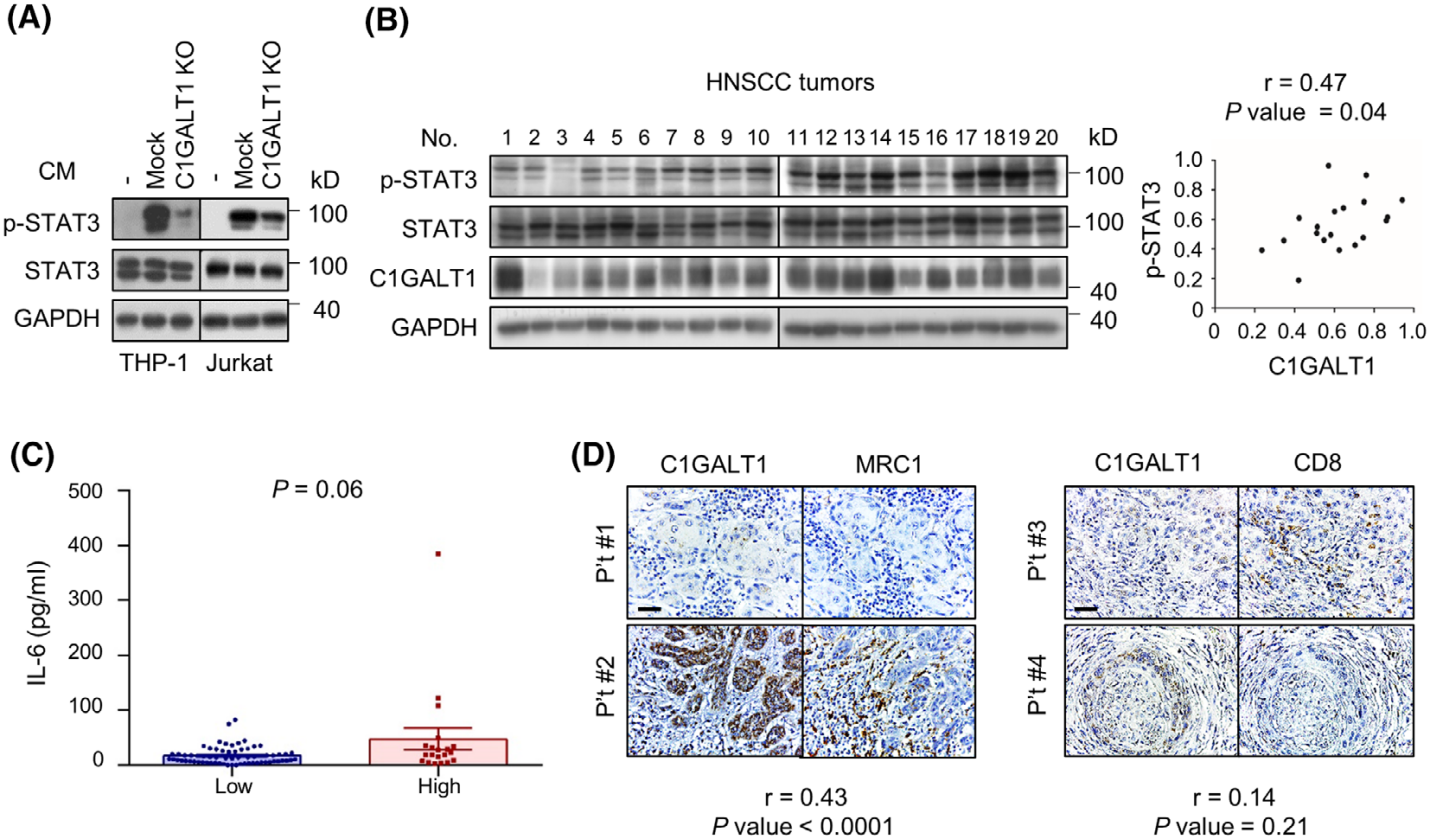
Correlation of C1GALT1 expression with p‐STAT3, tumor‐infiltrating macrophages, and CTLs in primary HNC tumors. (A) p‐STAT3 expression in THP‐1 and Jurkat cells. THP‐1 and Jurkat cell were starved for 24 h and then stimulated with the CM of Mock or C1GALT1 knockout (KO) SAS cells for 10 min. (−) indicates cells without treatment with CM. p‐STAT3 and STAT3 were evaluated using western blot analysis. GAPDH was used as an internal control. Experiments were performed in triplicate. (B) Expression of p‐STAT3, STAT3, and C1GALT1 in primary HNC tumors. Tissue lysates from primary HNC tumors (*n* = 20) were subjected to western blot analysis. Signals of p‐STAT3 and C1GALT1 were quantified using the imagej software. Pearson's correlation analysis was performed to evaluate the correlation between p‐STAT3 and C1GALT1. This experiment with human samples was performed once. (C) Correlation of C1GALT1 expression in HNC tumors with the IL‐6 levels in patient sera (*n* = 87). Expression of C1GALT1 was evaluated via IHC and recorded using a semi‐quantification system. Tumors were then designated into C1GALT1‐low or ‐high group. Levels of IL‐6 in patient sera were measured via ELISA. The differences in IL‐6 levels and C1GALT1 scores were evaluated using Mann–Whitney *U* test. This experiment with human samples was performed once. Data are presented as mean ± SEM. (D) Correlation of C1GALT1 expression with MRC1 and CD8 in primary HNC tumors (*n* = 87). Scale bars, 50 μm. MRC1, CD8, and C1GALT1 expression was evaluated via IHC and recorded in a semi‐quantification system (scores 0–3). The correlation of C1GALT1 expression with MRC1 or CD8 was evaluated via Spearman's rank correlation analysis.

To evaluate whether immune cells can sense the reduced IL‐6 expression in tumor cells, we treated THP‐1 and Jurkat cells with conditioned media from Mock or C1GALT1 knockout SAS cells. The results showed that the levels of p‐STAT3 were reduced in cells treated with the conditioned media from C1GALT1 knockout cells compared with those in the Mock cells (Fig. 5A). Consistently, the expression of p‐STAT3 was positively correlated with the levels of C1GALT1 in primary HNC tumors (Fig. 5B). The correlation between serum IL‐6 levels and C1GALT1 expression in tumors was evaluated using paired archival sera and primary tumors from HNC patients. The results showed that the patients with tumors expressing high levels of C1GALT1 had marginally higher serum IL‐6 levels (Fig. 5C). Consistent with our syngeneic mouse models, increased numbers of MRC1^+^ macrophages were observed in tumors with high C1GALT1 expression compared with those in tumors with low C1GALT1 expression (Fig. 5D, left). However, the number of CD8^+^ lymphocytes did not significantly correlate with C1GALT1 expression in the tumors (Fig. 5D, right). Overall, we demonstrate that, in clinical samples, the expression of C1GALT1 positively correlates with the levels of p‐STAT3 in tumors and IL‐6 levels in the sera of HNC patients. Moreover, in the TME, the population of MRC1^+^ macrophages was positively correlated with C1GALT1 expression.

### 3.6 Immune responses provoked by O‐glycan‐truncated tumor cells are phenocopied by treatment with an anti‐IL‐6R antibody

**Fig. 6:**
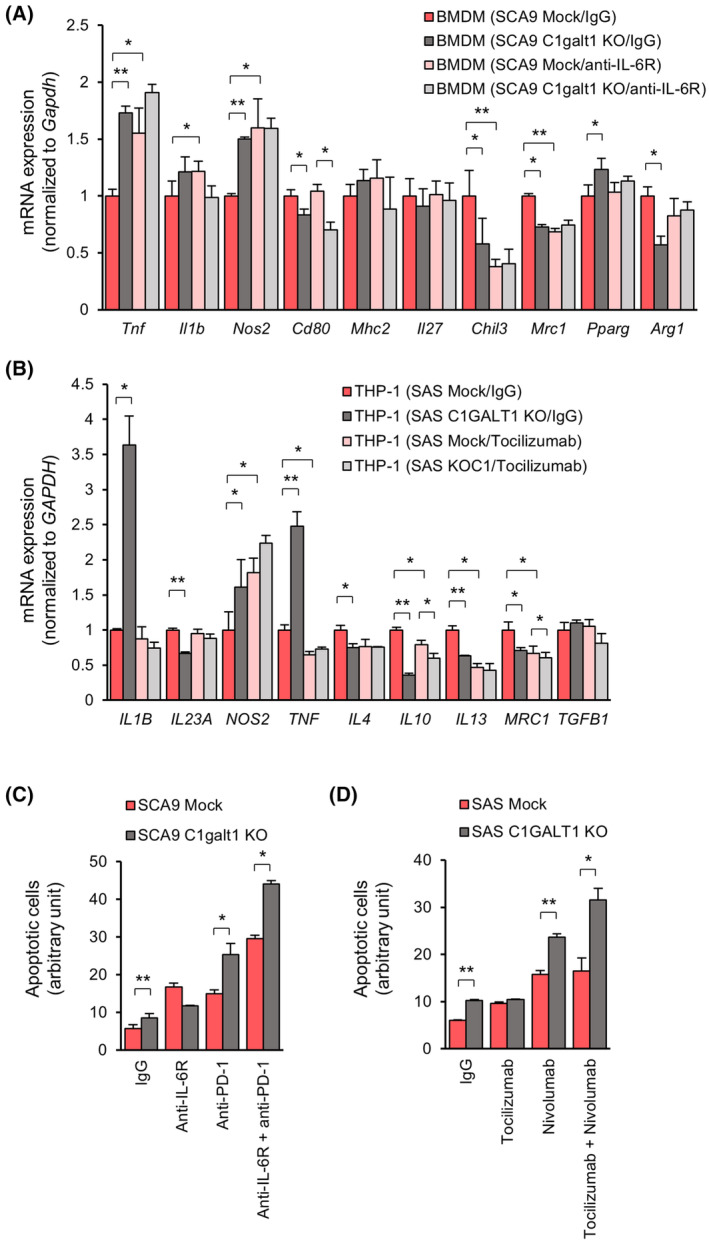
Effects of O‐glycan truncation in tumor cells on macrophages and CTL activities are phenocopied by blockade of the IL‐6/IL‐6R pathway *ex vivo*. (A) Expression of macrophage differentiation markers in the BMDMs incubated with control IgG or anti‐IL‐6 antibody and then cocultured with SCA9 cells. The differentiated BMDMs were incubated with 20 μg·mL^−1^ control IgG or anti‐IL6 antibody for 30 min and then cocultured with Mock or C1galt1 knockout (KO) SCA9 cells in transwells for another 24 h. BMDMs were harvested, and macrophage differentiation markers were evaluated using real‐time RT‐PCR analysis. Experiments were performed in triplicate. Data are presented as mean ± SD in arbitrary units. *P < 0.05 and **P < 0.01, analyzed using Student's *t*‐test. (B) Macrophage differentiation markers in the THP‐1 cells incubated with control IgG or Tocilizumab and then cocultured with SAS cells. Differentiated THP‐1 cells were incubated with 20 μg·mL^−1^ control IgG or Tocilizumab for 30 min and then cocultured with Mock or C1GALT1 KO SAS cells in transwells for another 24 h. Macrophage differentiation markers in THP‐1 cells were evaluated via real‐time RT‐PCR analysis. Experiments were performed in triplicate. Data are presented as mean ± SD in arbitrary units. *P < 0.05 and **P < 0.01, analyzed using Student's *t*‐test. (C) Effects of IL‐6R and PD‐1‐blocking antibodies on CTL‐mediated killing of Mock and C1galt1 KO SCA9 cells. CD3^+^/CD8^+^ cells were isolated from splenocytes and activated with mIL‐2 and CD3/CD28 Dynabeads. CTLs were incubated with 20 mg·mL^−1^ IgG, anti‐IL‐6R, anti‐PD‐1, or anti‐IL‐6R + anti‐PD‐1 antibodies for 30 min and then cocultured with wild Mock or C1galt1 KO SCA9 cells for another 24 h. CTLs were washed and removed, and apoptotic SCA9 cells were evaluated using fluorescence microscopy. Apoptotic cells were quantified using the imagej software. Experiments were performed in triplicate. Data are presented as mean ± SD in arbitrary units. *P < 0.05 and **P < 0.01, analyzed using Student's *t*‐test. (D) Effects of IL‐6R and PD‐1 blocking antibodies on T cell‐mediated killing of Mock and C1GALT1 KO SAS cells. Activated Jurkat cells were incubated with 20 μg·mL^−1^ IgG, tocilizumab, nivolumab, or tocilizumab + nivolumab for 30 min and then cocultured with Mock or C1GALT1 KO SAS cells for another 8 h. Apoptotic SAS cells were evaluated via fluorescence microscopy and quantified using the imagej software. Experiments were performed in triplicate. Data are presented as mean ± SD in arbitrary units. *P < 0.05 and **P < 0.01, analyzed using Student's *t*‐test.

O‐glycan truncation in tumor cells suppressed IL‐6 expression and induced pro‐inflammatory phenotypes in macrophages and CTLs. To further clarify whether these phenotypes were regulated by IL‐6 in tumors, an *ex vivo* coculture assay was performed along with anti‐IL‐6R antibody treatment. The macrophages preincubated with an anti‐IL‐6R antibody showed M1 polarization similar to those induced by the O‐glycan‐truncated tumor cells (Fig. 6A,B). Overexpression of wild‐type IL‐6 as well as mutant IL‐6 T166A suppressed M1 polarization of THP‐1 cells (Fig. S7). Treatment with the anti‐IL‐6R antibody enhanced the T cell‐mediated cytotoxicity in Mock cells to a level similar to that observed in O‐glycan‐truncated cells. In addition, treatment with anti‐PD‐1 antibodies further enhanced the CTL‐mediated cytotoxicity, particularly in the O‐glycan‐truncated tumor cells (Fig. 6C,D). Overall, blockade of the IL‐6/IL‐6R pathway phenocopied O‐glycan truncation in tumor cells *ex vivo*. Moreover, the maximum killing by CTLs is observed in O‐glycan‐truncated tumor cells treated with both anti‐IL‐6R and anti‐PD‐1 antibodies.

### 3.7 O‐glycosylation inhibitor itraconazole and anti‐PD‐1 antibody effectively suppress tumor growth *in vivo*

**Fig. 7:**
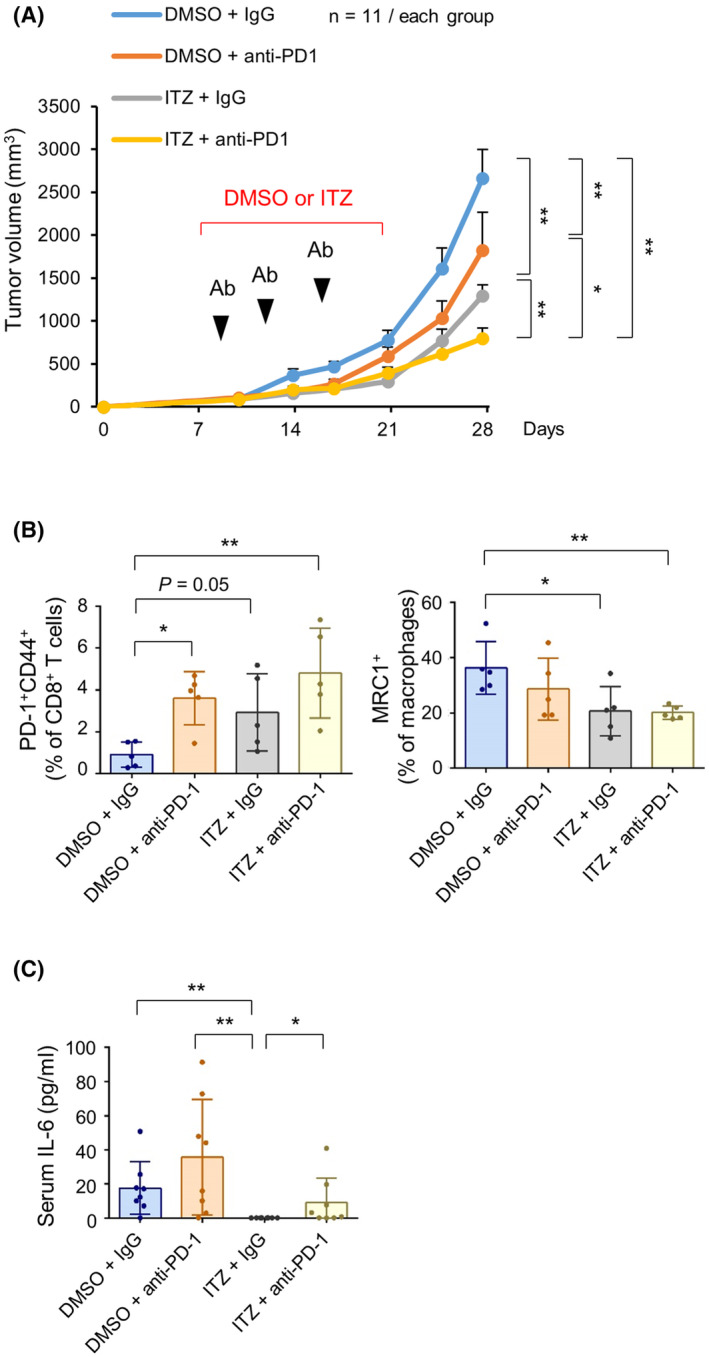
Targeting O‐glycosylation enhances the effects of anti‐PD‐1 immunotherapy in syngeneic mouse models. (A) Growth curves of MTC‐Q1 tumors treated with DMSO or itraconazole (ITZ) and rat IgG2a (IgG) or anti‐PD‐1 antibody. MTC‐Q1 cells (2 × 10^6^) were subcutaneously injected into the flanks of C57BL/6 mice (*n* = 11 in each group). DMSO or ITZ (600 mg·kg^−1^, intraperitoneal) was given daily from day 7 to 21. IgG or anti‐PD‐1 antibody (15 mg·kg^−1^, intraperitoneal) was injected at days 8, 11, and 15. Tumor volumes were recorded as 1/2 (Length × Width^2^). Data are presented as mean ± SEM. *P < 0.05 and **P < 0.01, analyzed using two‐tailed Mann–Whitney *U* test. (B) Percentages of PD‐1^+^CD44^+^CD8^+^ T cells and MRC1^+^ macrophages in tumors. Mice were sacrificed at day 28. Tumor‐infiltrating leukocytes were isolated and subjected to flow cytometry with antibodies against CD45, CD3, CD8, PD‐1, F4/80, and MRC1. Due to small tumor sizes in treatment groups, 5 mice per group were obtained. Data are presented as mean ± SEM. *P < 0.05 and **P < 0.01, analyzed using two‐tailed Mann–Whitney *U* test. (C) Serum IL‐6 levels. Serum samples (50 μL) were collected (*n* = 8/each group) from the submandibular vein at day 27. IL‐6 levels were measured using Sandwich ELISA. This experiment was performed once. Data are presented as mean ± SEM. *P < 0.05 and **P < 0.01, analyzed using Mann–Whitney‐*U* test.

To evaluate the effect of the combination of the O‐glycosylation inhibitor itraconazole and anti‐PD‐1 therapy *in vivo*, Mock MTC‐Q1 cells were subcutaneously injected into C57BL/6J mice (*n* = 11 for each group). Treatment with itraconazole and anti‐PD‐1 antibody was initiated 1 week after tumor inoculation. The combination of itraconazole with anti‐PD‐1 antibody was the most effective at tumor suppression compared with the control or single‐agent therapies (Fig. 7A). Tumor‐infiltrating leukocytes were evaluated, and the gating strategy was shown in Fig. S8. The results showed an increased number of PD‐1^+^CD44^+^CD8^+^ T cells in tumors treated with the anti‐PD‐1 antibody, itraconazole, or both (Fig. 7B, left). A reduced population of MRC1^+^ TAMs was observed in itraconazole‐ and itraconazole/anti‐PD‐1 antibody‐treated tumors (Fig. 7B, right). In addition, in mice treated with itraconazole alone, levels of serum IL‐6 were significantly suppressed (Fig. 7C). Suppression of viability by itraconazole was less dramatic *in vitro* compared with its suppressive effect on tumor growth *in vivo* (Fig. S9A). Complete blood counts were evaluated, and the results showed a decreased number of white blood cells in mice treated with anti‐PD‐1 antibody, itraconazole, or both, compared with the control group (Fig. S9B). Moreover, treatment of itraconazole and anti‐PD‐1 antibody decreased neutrophil and lymphocyte counts, respectively. Collectively, we show that targeting the tumor O‐glycosylation reshapes the TME and enhances the effects of anti‐PD‐1 therapy *in vivo*.

## 4 Discussion

**Fig. 8:**
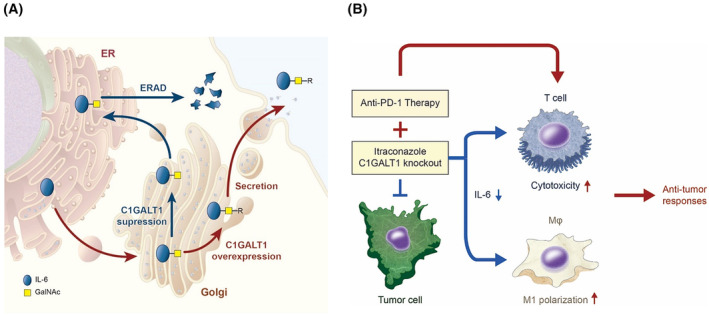
Targeting tumor O‐glycosylation reduces IL‐6 expression and enhances the effect of anti‐PD‐1 immunotherapy in HNC. (A) C1GALT1 modifies O‐glycans on IL‐6 in the Golgi apparatus. IL‐6 with proper O‐glycans is secreted to the extracellular space. Failure of O‐glycan modification by C1GALT1 causes degradation of IL‐6 through the ERAD pathway. (B) IL‐6 suppresses M1 polarization of macrophages and the activities of CTLs. Inhibition of C1GALT1 with itraconazole or knockout of C1GALT1 with CRISPR/Cas9 increases the antitumor responses of macrophages and CTLs, which in turn enhances the efficacy of anti‐PD‐1 immunotherapy in HNC.

Previously, we reported that overexpression of C1GALT1 in tumors predicts poor survival for patients with HNC [26]. In this study, we demonstrated that, being a crucial enzyme for O‐glycan elongation, C1GALT1 not only directly promotes cell survival and invasion but also facilitates tumor‐mediated immune evasion. Our data showed that C1GALT1‐mediated O‐glycosylation governs the immunosuppressive state of the TME partly through the IL‐6/IL‐6R pathway. IL‐6 undergoes O‐glycosylation at T166 in the Golgi apparatus and is secreted to the extracellular space. Proper O‐glycosylation of IL‐6 by C1GALT1 is attributed to its stability. Failure of O‐glycan extension in C1GALT1 knockout cells or upon itraconazole treatment resulted in an ERAD of IL‐6 and consequently reduced extracellular levels of IL‐6 (Fig. 8A). Targeting O‐glycosylation rescues the immunosuppressive TME and reinvigorates the function of macrophages and CTLs (Fig. 8B). The combination of itraconazole and anti‐PD‐1 therapy resulted in evident antitumor responses in mouse models. These results suggest that targeting tumor O‐glycosylation enhances the effects of anti‐PD‐1 immunotherapy by suppressing IL‐6 expression in HNC and is a promising strategy for remodeling the immunosuppressive TME.

Glycocodes in normal and transformed cells are deciphered by a wide variety of glycan‐binding receptors, called lectins, that are abundantly expressed by immune cells [39]. The altered cancer glycome actively engages in the crosstalk between tumors and immune cells, and targeting tumor‐associated glycosylation has been suggested to enhance antitumor immunity [40]. For example, blocking sialylation in tumor cells enhances the maturation of DCs and promotes the CD8 T cell‐mediated killing of tumor cells *in vivo* [41]. Sialoglycans are recognized as self‐associated molecular patterns (SAMPs) by Siglecs on T cells [42] and targeting the sialoglycan‐SAMP/Siglecs pathway results in an increased T cell activity [43]. Trastuzumab coupled with sialidase increases the antibody‐dependent cell‐mediated cytotoxicity against HER2‐expressing tumors [44]. The role of GalNAc‐type O‐glycans in the TME of HNC remains unclear. Here, we demonstrated that C1GALT1‐mediated O‐glycosylation contributes to the tumor‐induced immune tolerance by influencing the composition of tumor secretomes. Targeting O‐glycosylation suppresses tumor growth in syngeneic mouse models by reducing immunosuppressive macrophages and increasing tumor‐reactive T cells.

Although ICIs have shifted the paradigm in oncology treatment [45], complete responses are privileged to a minority of patients. Overcoming the resistance to ICI relies on rescuing T cell dysfunction and reprogramming the macrophages toward a tumoricidal status [46]. The combination of ICIs with immune‐modulating agents is the most promising strategy for the successful supression of tumor growth [47]. Our study showed that C1GALT1 inhibitor itraconazole suppressed tumor growth and introduced tumor‐reactive T cells into the TME. In addition, simultaneous administration of itraconazole with an anti‐PD‐1 antibody resulted in a high antitumor efficacy. Mechanistically, itraconazole not only blocks the O‐glycosylation‐mediated anti‐inflammatory effects but also provides additional antitumor effects by suppressing the EGFR pathways [26]. These results suggest that targeting O‐glycosylation could be a new avenue for rewiring a dysfunctional TME. It is worth mentioning that we observed significantly more MRC1^+^ cells in tumors with high C1GALT1 expression than in tumors with low C1GALT1 expression. However, the fact that C1GALT1‐low tumors can still grow and cause symptoms in patients suggests that other immune evasion mechanisms may be exercised in C1GALT1‐low tumors.

IL‐6 plays pivotal roles in lymphocyte differentiation, tissue inflammation, and tumor progression [23, 48, 49]. In patients with HNC, elevated serum IL‐6 levels predict recurrence and poor overall survival [50]. The association between IL‐6 and tumor immunology has been extensively studied in recent years. Elevation of IL‐6 levels in aged hosts dampens the antitumor immunity by impairing the Th1 differentiation of CD4^+^ T cells and subsequent activation of CD8^+^ T cells [51]. The Glioblastoma multiforme (GBM)‐derived IL‐6 promotes tumor growth by upregulating PD‐L1 expression in myeloid cells [52]. *IL‐6* knockout improves the efficacy of anti‐PD‐L1 blockade in the treatment of refractory leukemia [53]. Consistently, superior tumoricidal effects are also observed in GBM treated with anti‐IL‐6 and anti‐PD‐1 antibodies compared with monotherapy [54]. In this study, we observed elevated serum IL‐6 levels in patients with high C1GALT1 expression in tumors. Furthermore, we found that the inhibition of C1GALT1‐mediated O‐glycosylation suppressed IL‐6 expression. Blocking O‐glycan elongation or the IL‐6/IL‐6R axis induced tumoricidal phenotypes in macrophages and CTLs. Targeting O‐glycosylation by itraconazole successfully suppressed serum IL‐6 levels, upregulated tumor infiltrating CTLs, and downregulated MRC1^+^ TAMs *in vivo*. We provide evidence that IL‐6 is essential for maintaining an immunosuppressive TME and that suppressing IL‐6 expression by O‐glycan truncation can enhance the effect of anti‐PD‐1 therapy.

N‐glycosylation ensures proper protein folding, and abnormal glycosylation may cause misfolding of a protein, which is further recognized by ERAD mechanisms for turnover [55]. However, the impact of GalNAc‐type O‐glycosylation, which occurs in the Golgi apparatus, on protein stability is unclear. In this study, we showed that the loss of C1GALT1‐mediated O‐glycosylation resulted in reduced expression of intracellular IL‐6. Inhibition of ERAD or retrograde transportation to the ER restored IL‐6 levels in the C1GALT1 knockout cells. Deciphering the function of site‐specific O‐glycosylation is difficult because of the lack of consensus sequences. In this study, we found that the O‐glycosite at T166 controls the stability of IL‐6. These results suggest that the post‐ER quality control of IL‐6, and possibly many other proteins, is regulated by O‐glycosylation.

## 5 Conclusions

In summary, we showed that the tumor glycome actively participates in the crosstalk between cancer cells and immune cells. C1GALT1‐mediated O‐glycosylation is one of the glycocodes used by cancer cells to establish an immuno‐privileged environment that favors tumor progression. To our knowledge, this is the first study to demonstrate that targeting O‐glycosylation via C1GALT1 knockout or itraconazole reinvigorates the immunosuppressive milieu and enhances antitumor immunity by ICIs.

## Conflict of interest

The authors declare no conflict of interest.

## Author contributions

M‐CL is responsible for experimental design, analysis and interpretation of data, and manuscript writing. Y‐TC, H‐YW, C‐LH, and N‐YL contribute to development of methodology and acquisition of data. M‐CH and P‐JL contribute to study supervision, writing, review, and revision of the manuscript.

## Supporting information


**Fig. S1.** Cell viability and apoptosis of Mock and C1galt1 KO cells.
**Fig. S2.** Residual *Cas9* expression check‐up and tumor‐infiltrating leukocytes analysis.
**Fig. S3.** Real‐time RT‐PCR analysis of M1 and M2 markers expressed by primary BMDMs cocultured with SCA9 cells treated with DMSO or itraconazole (ITZ).
**Fig. S4.** Functional maps of C1GALT1‐regulated genes.
**Fig. S5.** IL‐6 expression post‐translationally regulated by C1GALT1.
**Fig. S6.** Electron‐transfer dissociation mass spectrometry (ETD/MS) of the O‐glycosylation site on IL‐6.
**Fig. S7.** Effect of mutant IL‐6 on differentiation of THP‐1 cells.
**Fig. S8.** Gating strategy for flow cytometry analysis of the tumor‐infiltrating leukocytes (TIL).
**Fig. S9.** Effect of itraconazole (ITZ) on cell viability *in vitro* and complete blood counts of mice with different treatments.

**Table S1.** Antibodies.

**Table S2.** Primer sequences.

## Data Availability

Microarray data are accessible under GEO Series accession number GSE116418.
