# Supplementary material for: Targeting tumor O‐glycosylation modulates cancer–immune‐cell crosstalk and enhances anti‐PD‐1 immunotherapy in head and neck cancer
Source: Mol Oncol. 2023 Jul 24;18(2):350–68. doi: 10.1002/1878-0261.13489 (PMC10850803; doi:10.1002/1878-0261.13489)
Supplement: Supplementary file 1 — Fig. S1. Cell viability and apoptosis of Mock and C1galt1 KO cells. Fig. S2. Residual Cas9 expression check‐up and tumor‐infiltrating leukocytes analysis. Fig. S3. Real‐time RT‐PCR analysis of M1 and M2 markers expressed by primary BMDMs cocultured with SCA9 cells treated with DMSO or itraconazole (ITZ). Fig. S4. Functional maps of C1GALT1‐regulated genes. Fig. S5. IL‐6 expression post‐translationally regulated by C1GALT1. Fig. S6. Electron‐transfer dissociation mass spectrometry (ETD/MS) of the O‐glycosylation site on IL‐6. Fig. S7. Effect of mutant IL‐6 on differentiation of THP‐1 cells. Fig. S8. Gating strategy for flow cytometry analysis of the tumor‐infiltrating leukocytes (TIL). Fig. S9. Effect of itraconazole (ITZ) on cell viability in vitro and complete blood counts of mice with different treatments. [file MOL2-18-350-s003.pdf]

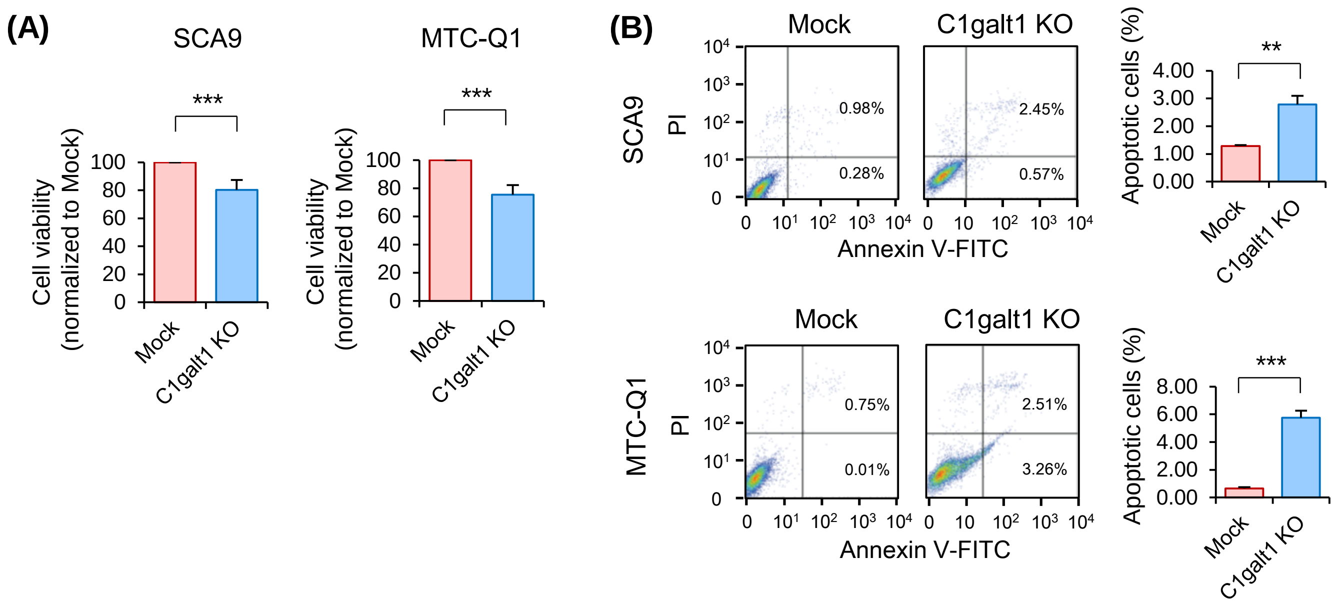

Figure S1. Cell viability and apoptosis of Mock and C1galt1 KO cells. (A) Cell viability of Mock or C1galt1 knockout (KO) SCA9 and MTC-Q1 cells. Cells (3× 10^3) were seeded into one well of a 96-well plate. Viability was measured at 72 h using alamarBlue™ assays. Data shown are representative of three independent experiments and are presented as mean ± SD. ****P* < 0.001, analyzed using a two-tailed Student's *t*-test. (**B**) Apoptosis assay. Mock or C1galt1 KO SCA9 and MTC-Q1 cells (1 × 10^6) were detached and washed with PBS. After centrifugation, cell pellets were resuspended in 100 µl cocktail containing annexin V-FITC and propidium Iodide (PI) and incubated for 15 min, followed by flow cytometric analysis. Apoptotic cells shown are annexin V^+ cells, including early and late apoptosis. Data shown are representative of three independent experiments and are presented as mean ± SD. ***P* < 0.01 and ****P* < 0.001, analyzed using a two-tailed Student's *t*-test.

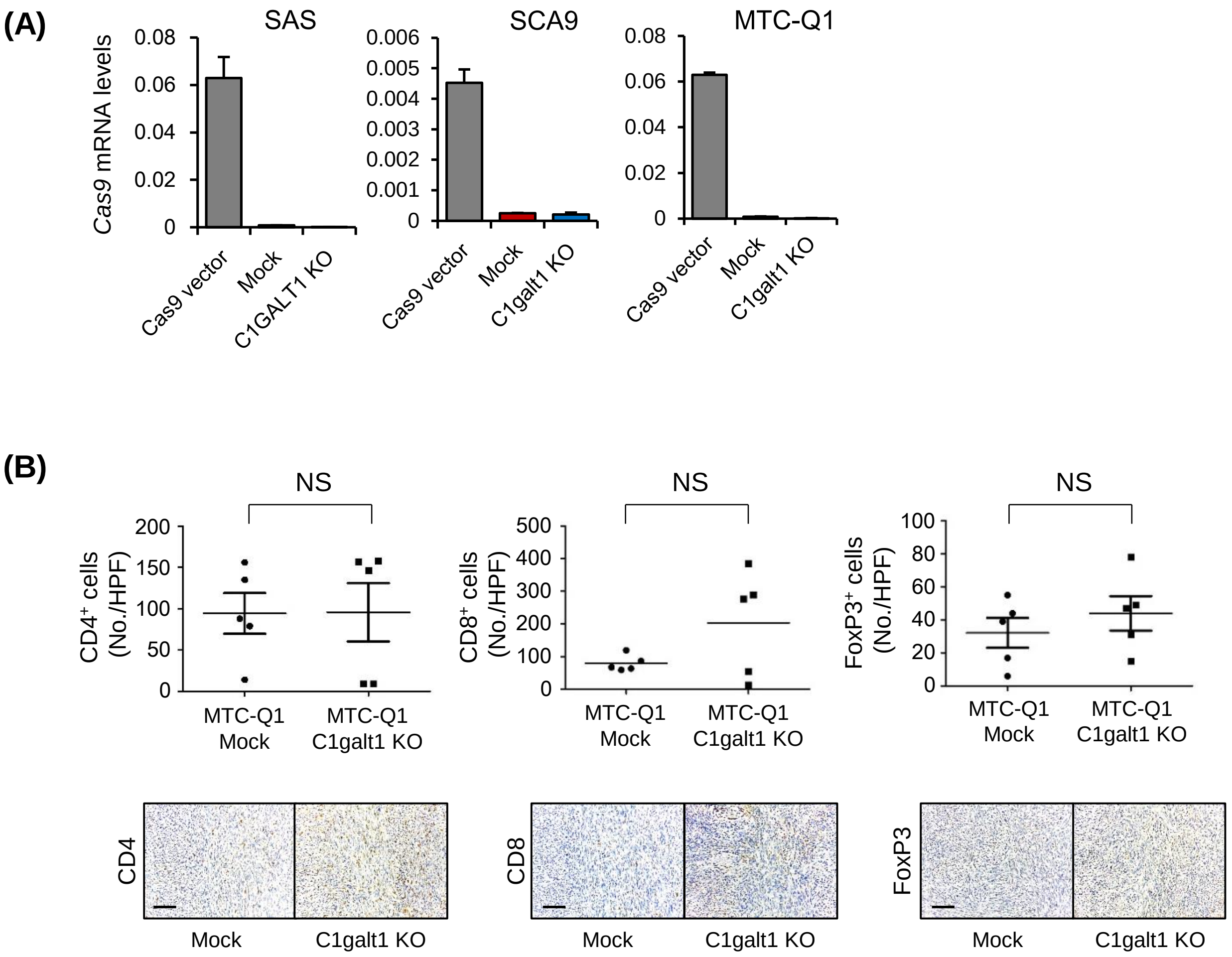

Figure S2. Residual *Cas9* expression check-up and tumor infiltrating leukocytes analysis. (**A**) Real-time RT-PCR analysis of *Cas9* expression in Mock and C1GALT1 KO cells. Mock cells transiently transfected with Cas9 vector containing the sequence of sgRNA against *C1GALT1* were used as positive controls. Data shown are representative of three independent experiments and presented as mean ± SD. (**B**) Numbers of tumor infiltrating leukocytes analyzed using immunohistochemistry (n = 5). CD4^{+} cells, CD8^{+} cells, and FoxP3^{+} cells were counted under high power fields of a microscope. Upper panels, numbers of positive cells presented by mean ± SEM. NS, not significant, analyzed using a two-tailed Student's *t*-test. Lower panels, representative images of CD4, CD8, or FoxP3 expression in Mock and C1galt1 KO tumors. Scale bars, 50 μm.

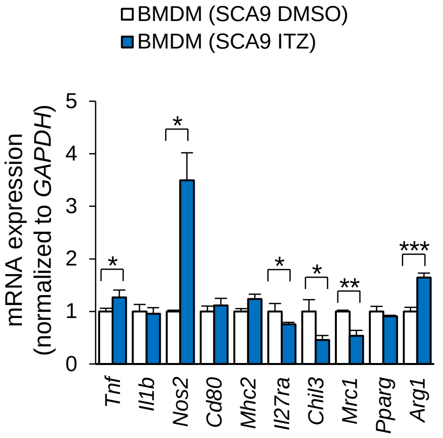

Figure S3. Real-time RT-PCR analysis of M1 and M2 markers expressed by primary BMDMs cocultured with SCA9 cells treated with DMSO or itraconazole (ITZ). Mouse primary BMDMs were harvested from bone marrows of Swiss Webster mice and incubated with L929 conditioned medium for 7 days to differentiate into M0 macrophages. SCA9 cells were treated with DMSO or 2.5 μM ITZ for 48 h and then cocultured with M0 macrophages, which were harvested 24 h later for real-time RT-PCR analysis. Data shown are representative of three independent experiments and presented as mean ± SD. $*P < 0.05$, $**P < 0.01$, and $***P < 0.001$ by Student's *t*-test.

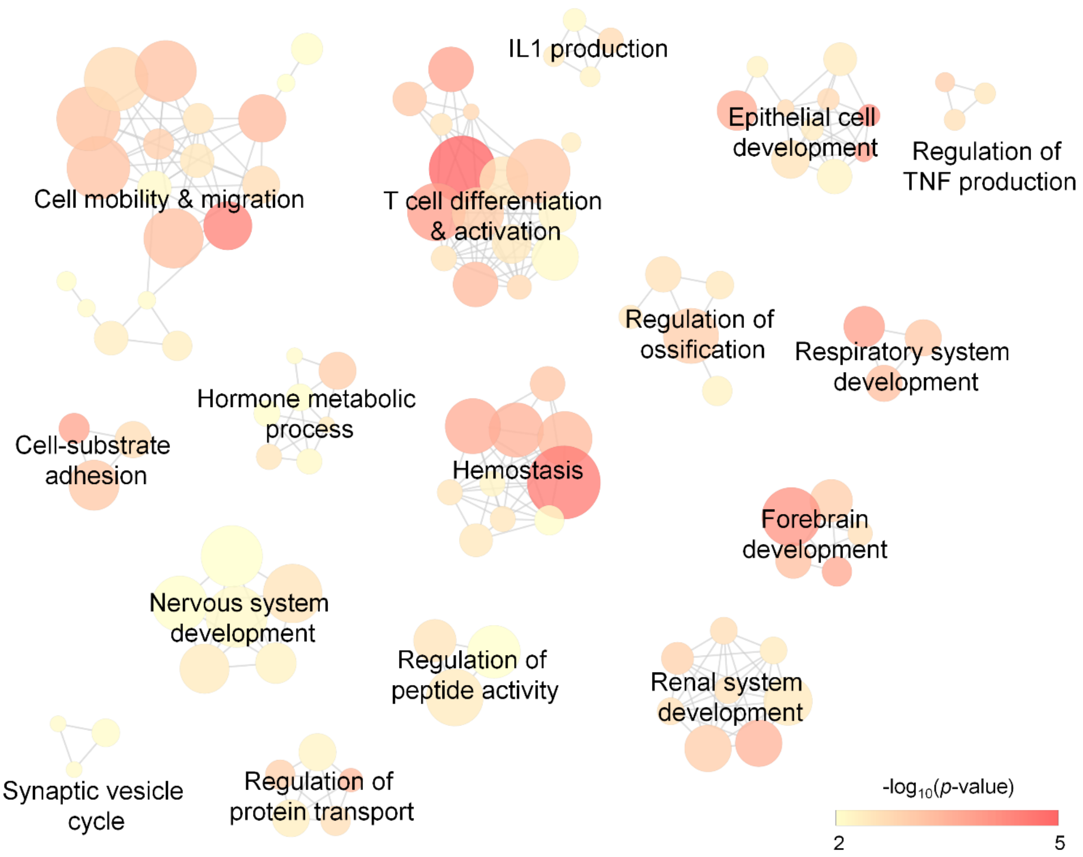

Figure S4. Functional maps of C1GALT1-regulated genes. SAS cells were transfected with non-targeting siRNA or siRNAs against *C1GALT1*. Total mRNA was analyzed using cDNA microarray analysis, and differentially expressed genes were further analyzed in this functional map. A node denotes the enriched GO term ($P < 0.01$) and an edge represents the gene overlap score between nodes > 0.5. Node color encodes the enriched *P*-value and node size is proportional to the number of genes which are associated with a given GO term. Groups of functionally related GO terms are manually identified and labelled with the appropriate terms.

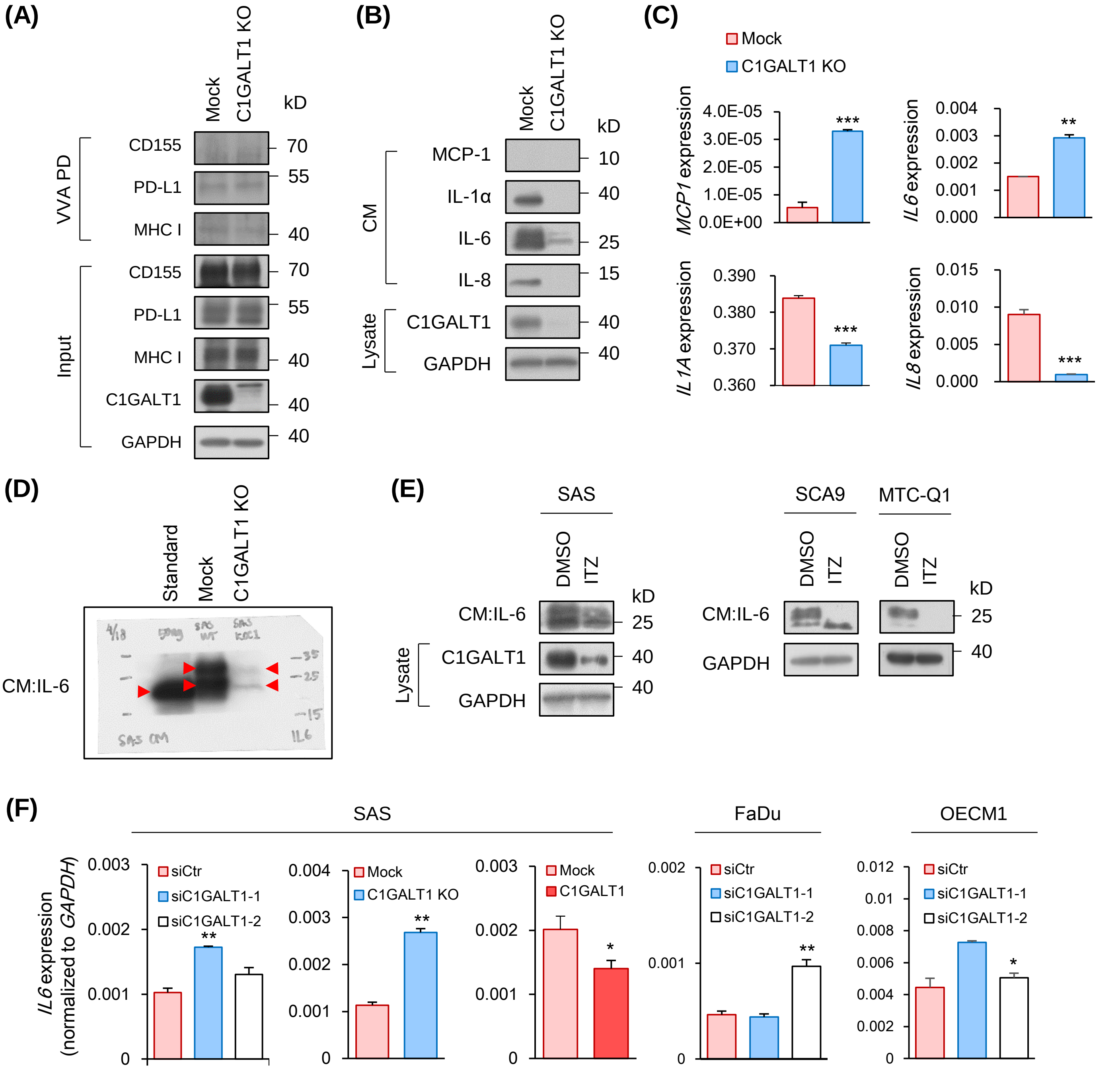

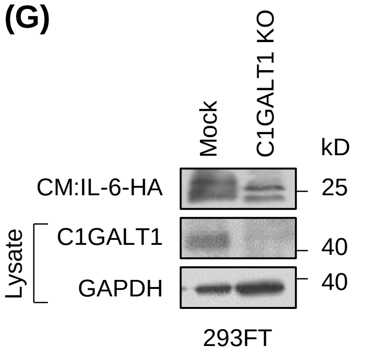

Figure S5. IL-6 expression post-translationally regulated by C1GALT1. (A) VVA pull-down assay and Western blot analysis of CD155, PD-L1, and MHC I in Mock or C1GLAT1 KO SAS cells. For the VVA pull-down (PD) assay, 500 μg of total protein was used. Input CD155, PD-L1, MHC I, C1GALT1, and GAPDH in cell lysates were shown in the lower panel. **(B)** Western blot analysis of MCP-1, IL-1α, IL-6, and IL-8 in the conditioned medium (CM) from Mock or C1GLAT1 KO SAS cells. C1GALT1 and GAPDH in cell lysates were shown in the lower panel. **(C)** Real-time RT-PCR of *MCP1, IL1A*, *IL6,* and *IL8* in Mock or C1GLAT1 KO SAS cells. Data shown are representative of three independent experiments and are presented as mean ± SD. $^{**}P < 0.01$ and $^{***}P < 0.001$ by Student's *t*-test.**(D)** Western blot analysis of standard IL-6 and IL-6 in the CM from Mock and C1GALT1 KO cells. Arrow heads indicate IL-6. **(E)** Western blot analysis of IL-6 in CM from DMSO or 2.5 μM itraconazole (ITZ) treated SAS, SCA9 or MTC-Q1 cells. C1GALT1 and GAPDH in cell lysates were shown in the lower panel. **(F)** Real-time RT-PCR analysis of *IL6* expression. C1GALT1 was knocked down, knocked out, or overexpressed in SAS cells. C1GALT1 was knocked down in FaDu and OEC-M1 cells. Data shown are representative of three independent experiments and presented as mean ± SD. $^{*}P < 0.05$ and $^{**}P < 0.01$ by Student's *t*-test. **(G)** Western blot analysis of ectopic HA-tagged IL-6 expression in the CM from Mock or C1GALT1 KO 293FT cells. Mock or C1GALT1 KO 293FT cells were transfected with *IL6-HA*/pcDNA3.1 for 48 h and then harvested for analysis. C1GALT1 and GAPDH in cell lysates were shown in the lower panel.

```
        10         20         30         40         50
MNSFSTSAFG PVAFSLGLLL VLPAAFPAPV PPGEDSKDVA APHRQPLTSS
        60         70         80         90        100
ERIDKQIRYI LDGISALRKE TCNKSNMCES SKEALAENNL NLPKMAEKDG
       110        120        130        140        150
CFQSGFNEET CLVKIITGLL EFEVYLEYLQ NRFESSEEQA RAVQMSTKVL
       160        170        180        190        200
IQFLQKKAKN LDAITTPDPT TNASLLTKLQ AQNQWLQDMT THLILRSFKE
       210
FLQSSLRALR QM
```

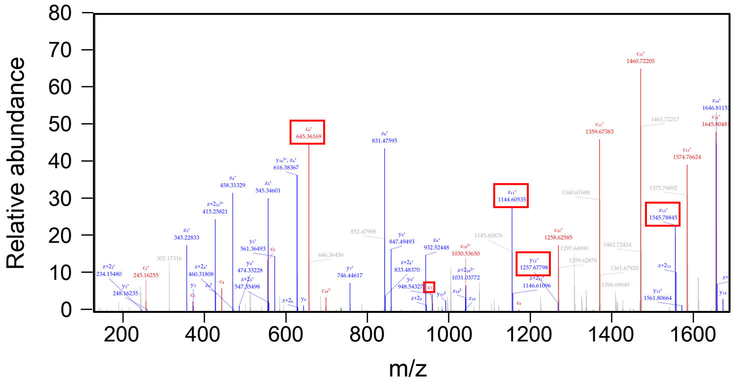

Figure S6. Electron-transfer dissociation mass spectrometry (ETD/MS) of the O-glycosylation site on IL-6. HA-tagged IL-6 was overexpressed in the C1GALT1 KO 293FT cells. IL-6 in the conditioned medium was purified using anti-HA agarose beads. Upper panel, the protein coverage map of IL-6. A HexNAc was identified on IL-6 at T166. Lower panel, tandem mass spectrum of the O-glycosylated peptide NLDAITT(166)PDPTTNASLLTK (red boxes). We analyzed two independent batches of IL-6 samples, and the O-glycosylation site was consistent between them.

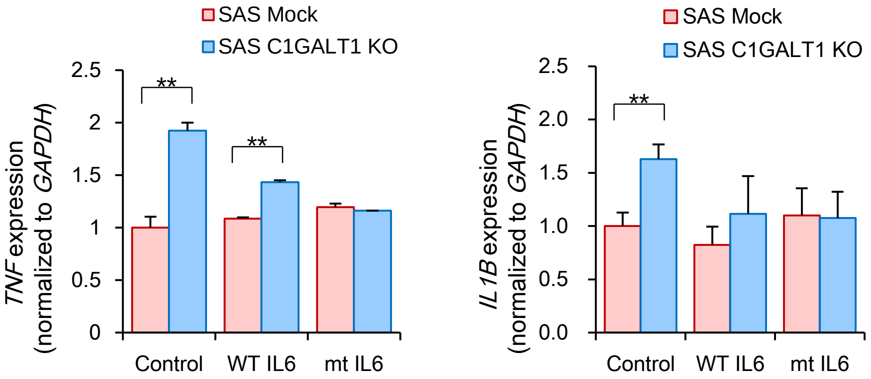

Figure S7. Effect of mutant IL-6 on differentiation of THP-1 cells. Mock or C1GALT1 KO SAS cells were transfected with *IL6/pcDNA3.1* (Mock IL-6) or *mtIL6 T166A/pcDNA3.1* (mt IL-6). Control cells were not transfected. THP-1 cells were differentiated into macrophages by incubation with 5 ng/mL PMA for 24 h. Macrophages were then cocultured with SAS cells in transwells for an additional 24 h. The expression of *TNF* and *IL1B* was evaluated by real-time RT-PCR analysis. Data shown are representative of three independent experiments and presented as mean ± SD. $^{**}P < 0.01$, analyzed using a two-tailed Student's *t*-test.

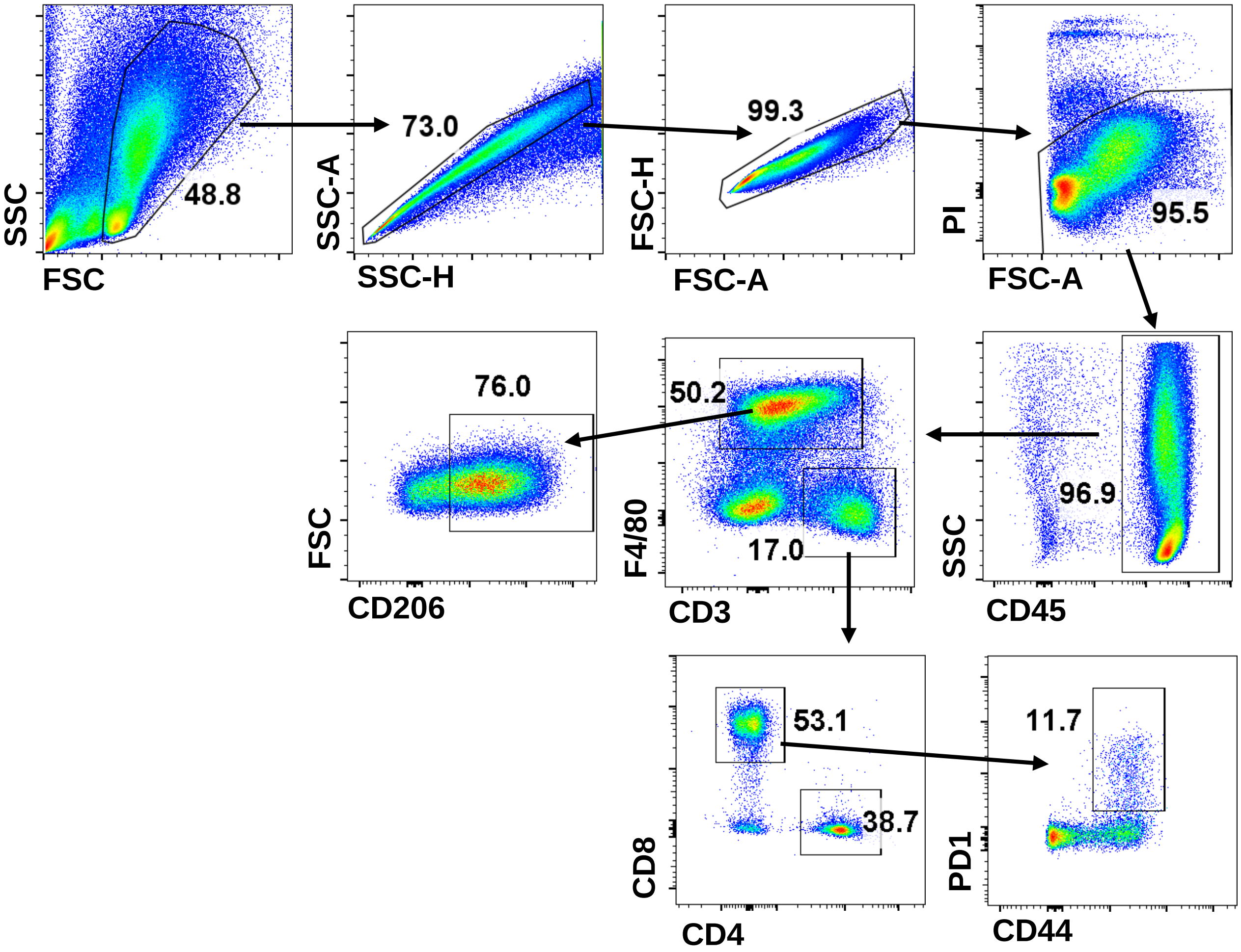

Figure S8. Gating strategy for flow cytometry analysis of the tumor-infiltrating leukocytes (TIL). After doublets and dead cells were excluded, macrophage and T cell populations were defined from total CD45^{+} TIL based on F4/80 and CD3 expression. The percentage of CD206^{+}F4/80^{+} tumor-associated macrophages and PD1^{+}CD44^{+}CD3^{+}CD8^{+} cytotoxic T cells were further determined.

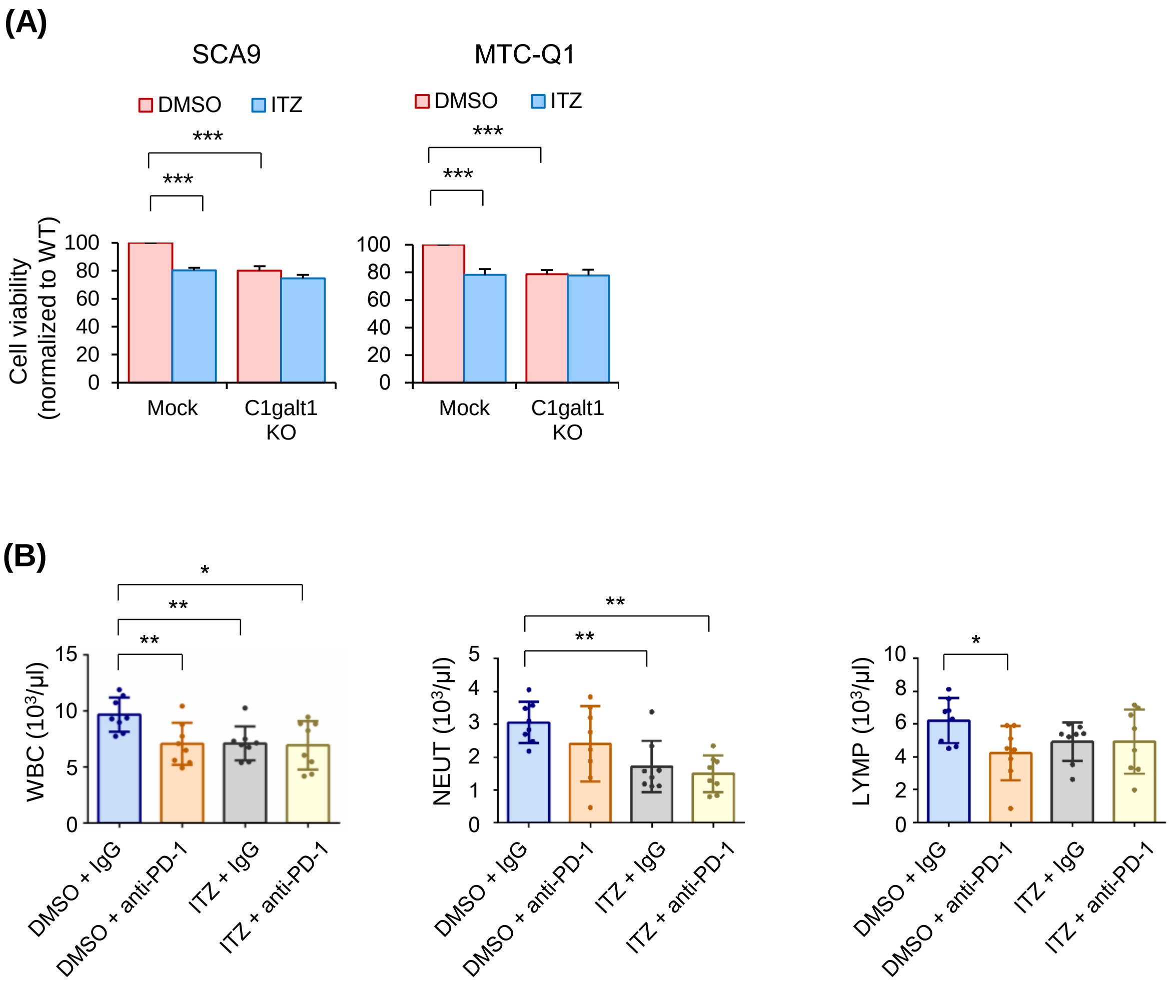

Figure S9. Effect of itraconazole (ITZ) on cell viability *in vitro* and complete blood counts of mice with different treatments. (A) Cell viability of Mock or C1galt1 KO SCA9 and MTC-Q1 cells treated with DMSO or ITZ. Cells (3×10^3) were seeded into one well of a 96-well plate on day 0. ITZ (10 µM for SCA9 cells and 1.25 µM for MTC-Q1 cells) was added at 24 h. Viability was measured at 72 h using alamarBlue™ assays. ****P* < 0.001, analyzed using a two-tailed Student's *t*-test. Data shown are representative of three independent experiments and presented as mean ± SD. **(B)** Blood samples (100 µl) were collected (n = 8 for each group) from the submandibular vein at 27 d. Complete blood counts were evaluated within 24 h of blood collection. WBC, white blood cell; NEUT, neutrophil; LYMP, lymphocytes. **P* < 0.05 and ***P* < 0.01. This experiment was performed once.
