# Supplementary material for: Targeting tumor O‐glycosylation modulates cancer–immune‐cell crosstalk and enhances anti‐PD‐1 immunotherapy in head and neck cancer
Source: Mol Oncol. 2023 Jul 24;18(2):350–68. doi: 10.1002/1878-0261.13489 (PMC10850803; doi:10.1002/1878-0261.13489)
Supplement: Supplementary file 2 — Table S1. Antibodies. [file MOL2-18-350-s002.docx]

Table S1. Antibodies

| **Antibody** | **Cat.** | **Company** | **Titer** |
| --- | --- | --- | --- |
| Flow cytometry | | | |
| APC-conjugated anti-mouse CD45 | 103111 | BioLegend | 1:100 |
| BV 421™-conjugated anti-mouse CD8a | 100737 | BioLegend | 1:100 |
| PE/Cy7-conjugated anti-mouse CD279 (PD-1) | 109109 | BioLegend | 1:100 |
| APC/Cy7-conjugated anti-mouse F4/80 | 123117 | BioLegend | 1:100 |
| PE-conjugated anti-mouse CD206 (MRC1) | 141705 | BioLegend | 1:100 |
| PerCP/Cy5.5-conjugated anti-mouse CD3 | 100218 | BioLegend | 1:100 |
| FITC-conjugated anti-mouse CD44 | 103021 | BioLegend | 1:100 |
| Western blot | | | |
| Anti-human C1GALT1 | sc-100745 | Santa Cruz Biotechnology | 1:1000 |
| Anti-human IL-6 | 12912 | Cell Signaling Technology | 1:1000 |
| Anti-human IL-6 | [MAB2061](https://www.rndsystems.com/products/human-il-6-antibody-1936_mab2061) | R&D systems | 1:1000 |
| Anti-PD-L1 | 13684 | Cell Signaling Technology | 1:1000 |
| Anti-CD155 | MAB2530 | R&D systems | 1:1000 |
| Anti-MHC I | sc-55582 | Santa Cruz Biotechnology | 1:1000 |
| Anti-human IL-8 | 94407 | Cell Signaling Technology | 1:1000 |
| Anti-human IL-1α | 84618 | Cell Signaling Technology | 1:1000 |
| Anti-human MCP1 | 81559 | Cell Signaling Technology | 1:1000 |
| Anti-human STAT3 | 12640 | Cell Signaling Technology | 1:1000 |
| Anti-human phospho-STAT3 | 9145 | Cell Signaling Technology | 1:1000 |
| Anti-mouse IL-6 | MAB406 | R&D systems | 1:1000 |
| Immunohistochemistry | | | |
| Anti-human C1GALT1 | sc-100745 | Santa Cruz Biotechnology | 1:500 |
| Anti-human MRC1 | 91992 | Cell Signaling Technology | 1:400 |
| Anti-human CD8 | 70306 | Cell Signaling Technology | 1:400 |
| Anti-mouse MRC1 | 24595 | Cell Signaling Technology | 1:400 |
| Anti-mouse granzyme B | 44153 | Cell Signaling Technology | 1:400 |
| Anti-mouse CD4 | [25229](https://www.cellsignal.com/products/primary-antibodies/cd4-d7d2z-rabbit-mab/25229) | Cell Signaling Technology | 1:400 |
| Anti-mouse CD8 | [98941](https://www.cellsignal.com/products/primary-antibodies/cd8a-d4w2z-xp-rabbit-mab/98941) | Cell Signaling Technology | 1:400 |
| Immunofluorescence | | | |
| Anti-human IL-6 | [MAB2061](https://www.rndsystems.com/products/human-il-6-antibody-1936_mab2061) | R&D systems | 1:1000 |
