# Supplementary material for: Targeting tumor O‐glycosylation modulates cancer–immune‐cell crosstalk and enhances anti‐PD‐1 immunotherapy in head and neck cancer
Source: Mol Oncol. 2023 Jul 24;18(2):350–68. doi: 10.1002/1878-0261.13489 (PMC10850803; doi:10.1002/1878-0261.13489)
Supplement: Supplementary file 3 — Table S2. Primer sequences. [file MOL2-18-350-s001.docx]

Table S2. Primer sequences

| Gene | Primer sequence (5’-3’) |
| --- | --- |
| mTnf | F: AAGAGGCACTCCCCCAAAAG |
|  | R: GTGGTTTGCTACGACGTGGG |
| mIl1b | F: AATGAAAGACGGCACACCCA |
|  | R: GCTTGTGCTCTGCTTGTGAG |
| mNos2 | F: GAAAACCCCTTGTGCTGTTCTC |
|  | R: ACCCCAAGCAAGACTTGGAC |
| mCd80 | F: GTTTCCCAAAGCCTCGCTTC |
|  | R: CTGACACGTGAGCATCTCCA |
| mMhc2 | F: CCAGGAAGAAGTCAGCCACAT |
|  | R: TCACTCCCAGGCCAGAAGATA |
| mIl27ra | F: CCCAGACTCTATTGGGATGG |
|  | R: GAACTGCTCCTGGGAACCTT |
| mChil3 | F: GAGACCATGGCACTGAACG |
|  | R: GTCTGAAAGACAAGAACACTGAG |
| mMrc1 | F: ACAGCTCATCATTTGGCT |
|  | R: CCACAGCATTGAGGAGTTTG |
| mPparg | F: GGGGGTGATATGTTTGAACTT |
|  | R: GAAAGACAACGGACAAATCACC |
| mArg1 | F: GAATCCTGGTACATCTGGGAA |
|  | R: GAATCTGCATGGGCAACC |
| IL1B | F: CAAACCTGACCCACCCAAGA |
|  | R: TGGCATTTTTGCGGCAGATG |
| IL23A | F: CCAGCTTCATGCCTCCCTAC |
|  | R: CTGGAGGCTGCGAAGGATTT |
| NOS2 | F: AGGTCCAAATCTTGCCTGGG |
|  | R: GCCTCTTTGAAGGAGCCGTA |
| TNF | F: TGCACTTTGGAGTGATCGGC |
|  | R: ACTCGGGGTTCGAGAAGATG |
| IL4 | F: CTTTGCTGCCTCCAAGAACACA |
|  | R: AGGAATCGGATCAGCTGCTTG |
| IL10 | F: GCCTTCAGCAGAGTGAAGACT |
|  | R: GGCAACCCAGGTAACCCTTA |
| IL13 | F: CACGGTCATTGCTCTCACTTG |
|  | R: GATGCTCCATACCATGCTGC |
| MRC1 | F: GGGAAAGGTTACCCTGGTGG |
|  | R: CAAGGAAGGGTCGGATCGTG |
| TGFB | F: AAGTGGACATCAACGGGTTCAC |
|  | R: TCCGTGGAGCTGAAGCAATA |
| IL8 | F: TTGTGTGGGTCTGTTGTAGG |
|  | R: CTTGGCCTCAATTTTGCTAT |
| IL1A | F: AGCTATGGCCCACTCCATGA |
|  | R: CCTTCCCGTTGGTTGCTACT |
| MCP1 | F: GAAAGTCTCTGCCGCCCTT |
|  | R: GGTGACTGGGGCATTGATTG |
| Cas9 | F: CAGATTCGCCTGGATGACCA |
|  | R: ATCCGCTCGATGAAGCTCTG |
